# Prevention of vascular calcification by the endogenous chromogranin A-derived mediator that inhibits osteogenic transdifferentiation

**DOI:** 10.1007/s00395-021-00899-z

**Published:** 2021-10-13

**Authors:** Setareh Orth-Alampour, Nathalie Gayrard, Silvia Salem, Shruti Bhargava, Vera Jankowski, Bernard Jover, Cécile Notarnicola, Heidi Noels, Emiel P. C. van der Vorst, Christoph Kuppe, Michael Wolf, Claudia Goettsch, Wendy Theelen, Heike Bruck, Danilo Fliser, Joseph Loscalzo, Zhuojun Wu, Nikolaus Marx, Walter Zidek, Àngel Argilés, Joachim Jankowski

**Affiliations:** 1 Institute of Molecular Cardiovascular Research, Medical Faculty, RWTH Aachen University, Pauwelsstrasse 30, 52074 Aachen, Germany; 2 RD-Néphrologie and EA7288, University of Montpellier, Montpellier, France; 3 Clinic for Pediatric Cardiology and Intensive Care, Medical School, Hannover, Germany; 4 Physiology and Experimental Medicine Heart Muscles, INSERM-CNRS-Université Montpellier, Montpellier, France; 5 Department of Nephrology, University Hospital Essen, Essen, Germany; 6 Helios Clinic Krefeld, Medical Clinic III, Krefeld, Germany; 7 Universitätsklinikum Homburg, Medizinische Klinik IV, Homburg, Germany; 8 Department of Medicine, Brigham and Women’s Hospital, Harvard Medical School, Boston, MA USA; 9 Medical Clinic I, Medical Faculty, RWTH Aachen University, Aachen, Germany; 10 Department of Nephrology, Meoclinic, Berlin, Germany; 11 Department of Nephrology, Charité, Berlin, Germany; 12 Experimental Vascular Pathology, Cardiovascular Research Institute Maastricht (CARIM), University of Maastricht, Maastricht, The Netherlands; 13 Institute of Experimental Medicine and Systems Biology, RWTH Aachen University, Aachen, Germany; 14 Department of Orthodontics, Medical Faculty, RWTH Aachen University, Aachen, Germany

**Keywords:** Vascular calcification, Adrenal glands, Vascular smooth muscle cell transdifferentiation, Cardiorenal syndrome

## Abstract

The adrenal glands participate in cardiovascular (CV) physiology and the pathophysiology of CV diseases through their effects on sodium and water metabolism, vascular tone and cardiac function. In the present study, we identified a new adrenal compound controlling mesenchymal cell differentiation that regulates osteoblastic differentiation in the context of vascular calcification. This peptide was named the “calcification blocking factor” (CBF) due to its protective effect against vascular calcification and is released from chromogranin A via enzymatic cleavage by calpain 1 and kallikrein. CBF reduced the calcium content of cells and thoracic aortic rings under calcifying culture conditions, as well as in aortas from animals treated with vitamin D and nicotine (VDN animals). Furthermore, CBF prevented vascular smooth muscle cell (VSMC) transdifferentiation into osteoblast-like cells within the vascular wall via the sodium-dependent phosphate transporter PIT-1 and by inhibition of NF-κB activation and the subsequent BMP2/p-SMAD pathway. Pulse pressure, a marker of arterial stiffness, was significantly decreased in VDN animals treated with CBF. In line with our preclinical data, CBF concentration is significantly reduced in diseases characterized by increased calcification, as shown in patients with chronic kidney disease. In preparation for clinical translation, the active site of the native 19-AS long native CBF was identified as EGQEEEED. In conclusion, we have identified the new peptide CBF, which is secreted from the adrenal glands and might prevent vascular calcification by inhibition of osteogenic transdifferentiation. The anti-calcific effects of CBF and short active site may therefore promote the development of new tools for the prevention and/or treatment of vascular calcification.

**Supplementary Information:**

The online version contains supplementary material available at 10.1007/s00395-021-00899-z.

## Introduction

Vascular calcification is an important component of cardiovascular (CV) pathology [59, 67, 71] that is highly prevalent in the general population increases with ageing [68] and is associated with increased mortality in patients with hypertension, chronic kidney disease (CKD) [12], and atherosclerosis [1, 15, 56]. The characteristics of calcified arteries include increased vascular stiffness and decreased elasticity. The pathogenesis of vascular calcification is complex and involves an imbalance of enhancers and inhibitors of the underlying processes. One essential step in the pathophysiology of vascular calcification is the transdifferentiation of vascular smooth muscle cells (VSMCs) into cells with an osteoblast-like phenotype. Although the understanding of arterial calcification has grown rapidly, only a limited number of vascular calcification regulators, such as osteopontin [70], osteoprotegerin [54, 78], as well as bone morphogenetic proteins [17, 80], have been identified. Most studies describe local paracrine regulatory mechanisms, such as the roles of bone morphogenetic proteins and Wnt signalling. These studies have mainly focused on the osteoblastic transdifferentiation of VSMCs under pathological conditions that result from the local imbalance between calcifying factors and inhibitors.

In this context, the adrenal glands are of particular interest, due to their involvement in the development and progression of cardiovascular disease through their effects on, among others, salt and water metabolism, vasoregulation and cardiac functions. In particular, the function of catecholamines in short-term vasoregulation is well known. Although specific regulators other than catecholamines have yet to be identified, considering the proteomic- and peptidomic-base identification of a large number of peptides and proteins produced by the adrenal medulla, it is possible that it contributes to long-term vascular adaptation by a yet undiscovered messenger [25, 34, 75]. So far, only a small number of these peptides, such as adrenomedullin and enkephalins, have been linked to endocrine or paracrine functions [13, 30]. While the role of catecholamines in short-term vasoregulation is well known, we hypothesized that the adrenal medulla also contributes to long-term vascular adaptation, e.g. osteoblastic transdifferentiation by yet undiscovered messengers.

In the present study, we aimed to identify an inhibitor of the vascular calcification process released by the adrenal glands. To this end, instead of traditional proteomic or peptidomic approaches, we used analytic mass spectrometry closely coupled with functional assays to focus specifically on identifying peptides relevant to vascular calcification, since the combination of chromatographic separation, screening of the chromatographic fractions by bioassays and identification of the molecular structure of the endogenous mediators has been proven in the past to be a highly successful approach to identify new cardiovascular and renal mediators (e.g. [35, 37, 61]). Such inhibitors of vascular calcification are of immense therapeutic value. While most prominently stage 5 CKD (also known as an end-stage renal disease (ESRD)) patients are at high risk to develop vascular calcification [63], patients suffering from diabetes, hypertension and advanced age are subpopulations with increased vascular calcification risk [11]. This correlation, along with the striking capacity of CBF to decrease vascular calcification in vivo*,* makes this peptide a clear candidate to explore a new approach in preventing or treating vascular calcification.

We report an adrenal factor with systemic effects that we named “calcification blocking factor” (CBF). Using in vitro*, *ex vivo and in vivo models, we show that CBF modulates VSMC transdifferentiation and thus vascular calcification, thereby participating in the balance between pro- and anti-calcifying mechanisms, and determine the pathways involved in its signal transduction. The level of CBF was found to be decreased in patients with CKD.

## Methods

### Mechanical disintegration and extraction

Fresh bovine adrenal glands were obtained from a slaughterhouse, and the glands were then mechanically pulverized and homogenized.

### Preparative reversed-phase chromatography

The supernatant of the homogenate was loaded onto a reversed-phase chromatography column. A 0.1% trifluoroacetic acid (TFA) solution in water was used as eluent A, and 80% acetonitrile in water was used as eluent B. Details of the chromatographic separation are given in the Supplementary method section.

### Effect of chromatographic fractions on ex vivo aortic vascular calcification

The thoracic region of the aorta of Wistar rats was used to analyse the effect of chromatographic fractions on calcification, since this region of the aorta shows a reproducible degree of calcification [31]. The thoracic aortic rings were incubated with a non-calcifying medium (NCM) and calcifying medium (CM) in the presence of adrenal gland chromatographic fractions for 7 days.

### Anion-exchange chromatography

The eluate was further fractionated by preparative anion-exchange chromatography. K_2_HPO_4_ in water was used as eluent A; NaCl in K_2_HPO_4_ was used as eluent B. Details of the chromatographic separation are given in the supplementary methods section.

### Reversed-phase chromatography

The eluate from the anion-exchange column was loaded onto a reversed-phase chromatographic column. A 0.1% TFA solution in water (1:1000, v/v; eluent A) was used for equilibration and 80% acetonitrile in water (80:20, v/v; eluent B), with a stepwise gradient of 20, 40, 60, 80, and 100% eluent B used for elution. Thereafter, the lyophilized fractions were resuspended and fractionated by a reversed-phase chromatographic column. Details of the chromatographic separation are given in the supplementary methods section. For details see the supplementary file.

### Matrix-assisted laser desorption/ionization mass spectrometry (MALDI-MS)

The lyophilized fractions from reversed-phase chromatography were analysed by matrix-assisted laser desorption/ionization mass spectrometry (MALDI-MS) and MALDI-TOF/TOF fragment ion analysis. Mass spectrometric measurements were performed on a Bruker Ultraflex-III TOF/TOF instrument (Bruker-Daltonic). Peptide identification using the obtained fragment ion mass data was performed using the Mascot search engine and the RapiDeNovo 3.0.1 sequencing tool. For details see the supplementary file.

### Electrospray ionization mass spectrometry (ESI–MS)

MS/MS fragmentation analysis was also performed by liquid chromatography/electrospray ionization-mass spectrometry (ESI–MS). Two capillary HPLC pumps with a micro-vacuum degasser, microwell-plate autosampler, and diode array multi-wavelength detector were used for chromatography. The mass spectrometric data were collected using the software HyStar 3.2 and analysed using the software Data Analysis 4.0 (Bruker-Daltonic). Glutathione was used as an internal standard to calculate the recovery rate. For details see the supplementary file.

### Peptide synthesis

CBF was synthesized automatically by the solid-phase method. Purification of the crude peptide was carried out by preparative HPLC on a PolyEncap A300 column in water with increasing concentrations of ACN as the mobile phase. An eluent gradient of 5–70 (v/v-%) ACN/water (0.1% TFA) was used. The peptide was characterized by MALDI-MS on a Voyager-DE STR BioSpectrometry Workstation MALDI-TOF mass spectrometer. For details see the supplementary file

### Isolation of adrenal chromaffin granules and stimulation with carbachol

Granules were isolated from fresh bovine adrenal glands obtained from a slaughterhouse using 0.32 mol L^−1^ sucrose and homogenized with an Ultra-Turrax homogenizer. After several centrifugation steps, the pellet was resuspended in water. To actively release CBF from the chromaffin granules, carbachol was added and PBS was used as a negative control. The supernatants were analysed by ESI-MS. For details see the supplementary file.

### Identification of the enzymes that cleave CBF from chromogranin A

The protease prediction tool Proteasix [40] was used to predict the enzymes likely responsible for cleaving CBF from chromogranin A. Enzyme candidates (kallikrein and calpain 1) were identified by entering protein ID P10645 as the input, with amino acid 358 as the N-terminal amino acid and amino acid 376 as the C-terminal amino acid of CBF. A peptide containing ten additional amino acids from chromogranin A at the N-terminus of CBF (LAKELTAEKR-LEGQEEEEDNRDSSMKLSF) and a peptide containing ten additional amino acids from chromogranin A at the C-terminus (LEGQEEEEDNRDSSMKLSF-RARAYGFRGP) were synthesized and used to identify the enzymes that cleave the N-terminal and C-terminal ends of CBF, respectively, from chromogranin A.

### Identification of post-translational modification of chromogranin A cleaving enzymes by matrix-assisted laser desorption/ionisation time of flight mass spectrometry

Post-translational modifications of chromogranin A cleaving enzymes were identified using matrix-assisted laser desorption/ionization-time of flight mass spectrometry (MALDI-TOF/TOF mass spectrometry) as previously described [36]. Briefly, proteins of 35 µg adrenal glands of C57BL/6J ApoE^−/−^ mice (bred and housed under specific pathogen-free conditions in the animal facility of RWTH Aachen university hospital; agreement number: 81-02.04.2017.A504) with and without chronic kidney disease (CKD) were isolated. The induction of CKD was performed by a 1.5-week diet of 0.3% adenine. A stable CKD condition was subsequently achieved with 0.15% adenine for 4.5 weeks. In addition, the animal received a high-fat diet during the whole time of the experiments starting 4 weeks before the induction phase. The induction and maintenance of CKD were monitored by analysis of plasma urea and creatinine levels.

The glands were then mechanically pulverized and homogenized and an equal amount of proteins were separated by 12% SDS–polyacrylamide gel electrophoresis. Proteins were stained using Coomassie Brilliant Blue G-250 (BioRad, Munich, Germany). Proteins corresponding to sizes of 75–120 kDa were separated and equilibrated using ammonium bicarbonate in acetonitrile. Proteins were trypsinized with ammonium bicarbonate (50 mmol L^−1^) and 0.03% w/v trypsin for 24 h at 37 °C. The resulting tryptic peptides were desalted and concentrated utilizing the ZipTip_c18_ technology (Millipore, Billerica, MA, USA) and water with 0.3% trifluoroacetic acid and eluted with 80% acetonitrile directly onto the MALDI target plate (MTP-Ground steel 400/384; Bruker-Daltonic) using alpha-cyano-4-hydroxycinnamic acid as matrix. The subsequent mass spectrometric (MS) analyses were performed using a MALDI-TOF/TOF mass spectrometer (Ultraflex III; Bruker-Daltonic, Germany). A database search (Swiss-Prot) using the Mascot 2.2 search engine (Matrix Science Inc., Boston, MA) and Bruker Bio-Tool 3.2 software (Bruker Daltonic, Bremen, Germany) was performed with the calibrated and annotated spectra to calculate the peptide mass signal for each entry into the sequence database; compare the experimental MALDI-MS and MALDI-MS/MS dataset, to assign a statistical weight to each peptide match using empirically determined factor [41]. For details see the supplementary file.

### In vitro post-translational oxidation of calpain 1

Calpain 1 (Sigma-Aldrich, Taufkirchen, Germany) (1 mg mL^−1^) was post-translationally oxidized in vitro by incubation with hydroperoxyphosphanone (HO_3_P) at 25 °C for 3 h. After extensive dialysis against PBS to remove the hydroperoxyphosphanone, the calpain 1 amount was assessed using western blot. Calpain 1 used as a control was treated in the same way but without the addition of hydroperoxyphosphanone. Oxidation status was assessed by mass spectrometry.

### Enzyme cleavage activity of native and post-translationally oxidized calpain 1

Native and in vitro post-translationally oxidized calpain 1 (1 mg mL^−1^) was incubated with elongated CBF in PBS buffer. Aliquots (5 µL) were time-dependently taken from the reaction solution, deproteinized and analysed by MALDI mass spectrometry as described above. The mass signal intensity of the CBF over the internal standard C^13^ angiotensin II (0.1 µg µL^−1^) was normalized and quantified.

### Cell culture and calcification induction in human aortic smooth muscle cells

Human aortic smooth muscle cells (HAoSMCs) of a male donor (58 years of age, Caucasian with diabetes type II) were purchased from Promocell (Heidelberg; Germany; Lot No. 411Z027.3) and cultivated in a smooth muscle cell medium. HAoSMCs of passages 4–9 were used. Dulbecco’s modified Eagle’s medium (DMEM) containing 25 mmol L^−1^ glucose supplemented with 2.5% foetal calf serum and 2.8 mmol L^−1^ phosphate was used as CM to induce calcification. The CM was supplemented with CBF or individual CBF fragments. DMEM containing 25 mmol L^−1^ glucose supplemented with 2.5% foetal calf serum and 0.9 mmol L^−1^ phosphate was used as a non-calcifying reference medium. HAoSMCs were incubated for 7 days. Details of cementoblasts are given in the supplementary methods section. For details see the supplementary file.

### Kinase activity profiling

Kinase activity profiling using PamChip^®^ Ser/Thr Kinase assay (STK; PamGene International, ′s-Hertogenbosch, The Netherlands) has been previously described [29]. Each STK-PamChip^®^ array contains 144 individual phospho-site(s) that are peptide sequences derived from substrates for Ser/Thr kinases. HAoSMCs were washed once in ice-cold PBS after the respective treatments, with three biological replicates per condition, and lysed for 15 min on ice using M-PER Mammalian Extraction Buffer containing Halt Phosphatase Inhibitor and EDTA-free Halt Protease Inhibitor Cocktail (1:10 each; Sigma-Aldrich). Lysates were centrifuged for 15 min at 16.000*g* at a temperature of 4 °C in a pre-cooled centrifuge. Protein quantification was performed with Pierce™ Coomassie Plus (Bradford) Assay according to the manufacturer’s instructions. For the STK assay, 0.5 µg of protein and 400 µmol L^−1^ ATP were applied per array (*N* = 3 per condition) together with an antibody mixture to detect the phosphorylated Ser/Thr. After a 1 h incubation at a temperature of 30 °C, where the sample was pumped back and forth through the porous material to maximize binding kinetics and minimize assay time, a second FITC-conjugated antibody was used to detect the phosphorylation signal. Imaging was performed using an LED imaging system, and the spot intensity at each time point was quantified using the BioNavigator software version 6.3 (PamGene International, ′s-Hertogenbosch). Upstream kinase analysis (UKA) [14] used as the functional scoring method was used to rank kinases derived from the combined specificity scores that were based on peptides linked to a kinase and acquired from six databases. Sensitivity scores were based on treatment–control differences.

### Ex vivo calcification of rat thoracic aortic rings

The thoracic region of the aorta of Wistar rats was used to analyse the effect of the identified peptides on calcification, since this region of the aorta shows a reproducible degree of calcification [31]. The endothelial layer of each thoracic aortic ring was manually damaged. The aortic rings were then incubated with NCM or CM supplemented with CBF or individual CBF fragments for 7 days. For details see the supplementary file.

### Transfection of human aortic smooth muscle cells

For siRNA transfection, hAoSMCs were detached and resuspended in a growth medium. Transfection was performed using the Neon Transfection System. PIT-1 siRNA with the sequence 5′-GCCGTAGTTTACAGTATTTAA-3′ was used to knock down PIT-1, and siRNA specific for GFP from Qiagen served as a control. 24 h after transfection, the growth medium was replaced with CM supplemented with CBF. NCM was used as a reference. The cells were incubated for 7 days. The transfection was repeated 3 days after incubation. For details see the supplementary file.

### Effect of CBF in an animal model with enhanced arterial calcification

An animal model of elastocalcinosis (VDN rats) in which increased arterial calcification has been demonstrated [50] was chosen to assay the effects of CBF in vivo. Three groups of 6-week-old Wistar rats were given regular rat chow and spring water ad libitum for 1 week before the experiments. Rats in the two groups received CBF or vehicles infused through an osmotic pump. CBF treatment was initiated 3 or 4 days before the induction of vitamin D_3_ and nicotine administration. Untreated rats served as control. Four weeks later, blood pressure and systolic, diastolic, mean arterial, and pulse pressures were determined. Each obtained aorta was cut into segments for further analysis. For details see the supplementary file

### Histological calcification staining and immunofluorescence staining

Formaldehyde-fixed thoracic aortic rings were used for histological and immunohistochemical staining. The rings were dehydrated and embedded in paraffin, and 5-µm slices were prepared by a microtome. The slices were deparaffinized and dehydrated before staining. von Kossa staining was performed to visualize the calcified areas of the aortic rings.

For immunohistochemical analyses, sections were blocked and incubated with primary antibodies at 4 °C overnight and with secondary antibodies at room temperature for 30 min to 1 h. Samples were visualized with a LEICA DM5500B microscope. Staining or protein expression was determined as the stained area using ImageJ software, and the corresponding results are expressed as a percentage of the total aortic area per section. For details see the supplementary file

### Measurement of the calcium and phosphate content

To measure the calcium and phosphate content, cells or thoracic aortic rings were washed with PBS and the aortic rings were dried and weighed. The dried aortic rings and cells were decalcified, and the calcium content in the supernatant after centrifugation was determined with the *o*-cresolphthalein complexone method according to the manufacturer’s protocol. The phosphate content in the supernatant was measured using a phosphate colorimetric kit (phosphate reacts with a chromogenic complex; Sigma-Aldrich, Taufkirchen, Germany) according to the manufacturer’s protocol. The protein content of the cells was measured by the bicinchoninic acid (BCA) protein assay method. The calcium and phosphate contents were normalized to the total protein content of the cells or the dry weight of the aortic rings. For details see the supplementary file.

### Reverse transcription quantitative polymerase chain reaction (RT-qPCR) analyses of mRNA expression in human aortic smooth muscle cells and vdn rats

Total RNA was extracted from hAoSMCs and aortic tissue using an RNeasy Mini Kit. Gene expression levels were quantified by real-time PCR using SYBR Green I dye chemistry on a LightCycler 480 system. PCR primers (Table 1) were designed using LightCycler Probe Design software 2.0. Expression levels relative to standard curves were determined with LightCycler analysis software (version 3.5). For details see the supplementary file.

**Table 1:** The primer sequences for RT-qPCR reaction

Target	Species	Sequence (5′–3′)
PIT-1-for	Human	AGCGTGGACTTGAAAGAGGA
PIT-1-rev	Human	TACAGGCCGGAATCCTTATG
PIT-2-for	Human	TCTCATGGCTGGGGAAGTTAGT
PIT-2-rev	Human	TTGCGACCAGTGAGAATCCTAT
BMP2-for	Human	GAGGTCCTGAGCGAGTTCGA
BMP2-rev	Human	ACCTGAGTGCCTGCGATACA
BMP2-for	Rat	ACAGCGGAAGCGTCTTA
BMP2-rev	Rat	CACAACCATGTCCTGATAGTTT
RUNX2-for	Human	CGCCATGACAGTAACCACAG
RUNX2-rev	Human	ACCATGGTGGAGATCATCG
RUNX2-for	Rat	CGCATTCCTCATCCCAGTAT
RUNX2-rev	Rat	TCTGTAATCTGACTCTGTCCTTGTG
OSX-for	Human	GCCAGAAGCTGTGAAACCTC
OSX-rev	Human	GCTGCAAGCTCTCCATAACC
OSX-for	Rat	CCTACTTACCCGTCTGACTTT
OSX-rev	Rat	GCCCACTATTGCCAACTG
MSX2-for	Human	AAGAAAACAGGGCTTGGTGCCTC
MSX2-rev	Human	GCGCAAGTTCCGTCAGAAACAG
MSX2-for	Rat	GGAGGCGGAACTGGAAA
MSX2-rev	Rat	ATAGAGTCCCACAGGCG
Osteocalcin-for	Human	CGATAGGCCTCGTGAAAGC
Osteocalcin-rev	Human	GGCAGCGAGGTAGTGAAGAG
SOX9-for	Human	AGCTCTGGAGACTTCTGAACGAGA
SOX9-rev	Human	ACTTGTAATCCGGGTCCTCCTTCT
ALP-for	Human	GGTGGAAGGAGGCAGAATTGA
ALP-rev	Human	GTCTTCCGAGGAGGTCAAGC
Collagen I-for	Human	TCTAGACATGTTCAGCTTTGTGGAC
Collagen I-rev	human	TCTGTACGCAGGTGATTGGTG
β-Actin-for	Human	CACCAACTGGGACGACAT
β-Actin-rev	Human	ACAGCCTGGATAGCAACG
RPLPO-for	Rat	CACTGGCTGAAAAGGTCAAGG
RPLPO-rev	Rat	GACTTGGTGTGAGGGGCTTA

### Western blot analysis of NF-κB activation

After stimulation of HAoSMCs with the corresponding condition, cells were lysed with RIPA (radioimmunoprecipitation assay) lysis buffer (50 mM tris–Cl (pH 7.6)) including EDTA-free Halt Protease Inhibitor Cocktail (1:10; Sigma-Aldrich) and Halt Phosphatase Inhibitor Cocktail (1:10; Sigma-Aldrich). The lysates were centrifuged at 13,000*g* at 4 °C for 15 min and the supernatants were collected. An equal amount of proteins from each sample was resolved by 10% SDS-polyacrylamide gel electrophoresis, transferred to nitrocellulose membranes, and blocked with 5% bovine serum albumin (BSA) for 1 h at room temperature. The blots were incubated with the monoclonal rabbit anti-phosphor-NF-κB (p56) antibody or monoclonal rabbit anti-NF-κB (p56) antibody (both Cell Signaling) 1:1000 at 4 °C overnight. The blots were subsequently incubated with horseradish peroxidase-conjugated anti-rabbit immunoglobulin G (IgG; Santa Cruz Biotechnology) at 1:10,000 for 1 h at room temperature. Immunoreactive bands were visualized via enhanced chemiluminescence, and densitometry was performed using Quantity One software (Bio-Rad Laboratories).

### Analyses of cell viability of the VSMCS

To determine the effect of CBF on the viability of the cells, hAoSMCs were seeded in a 96-well plate at a density of 1 × 10^4^ cells/well. The cells were incubated with NCM or CM without or supplemented with 100 nmol L^−1^ CBF for 4 days. After incubation, the cultivation medium was removed and cells were incubated with 12 mmol L^−1^ MTT (3-(4,5-dimethyl-2-thiazolyl)-2, 5-diphenyl-2H-tetrazolium bromide) solution for 40 min at 37 °C. After removing the solution, the cells were incubated with 50 µL of DMSO for 20 min at room temperature. The signal was measured using a fluorescence plate reader at an excitation wavelength of 540 nm and an emission wavelength of 630 nm.

### Enrichment of apoptotic bodies and flow cytometry

Apoptotic bodies were enriched by the centrifugation method. The hAoSMCs were seeded in a 12-well plate at a density of 5 × 10^5^ cells/well. The cells were incubated with NCM or CM without or supplemented with 100 nmol L^−1^ CBF for 5 days. Briefly, the apoptotic supernatant was centrifuged at 500*g* for 10 min to pellet cells, and the resulting supernatant was centrifuged at 3000*g* for 20 min to pellet apoptotic bodies. The apoptotic bodies were resuspended in annexin V binding buffer containing annexin V–FICT (1:500, BD Pharmingen) and incubated for 30 min at room temperature. Samples were pelleted at 3000*g* for 20 min, washed 1 × with PBS filtered with a 0.1 µm pore size hydrophilic polyethersulfone (PES) membrane (Millipore Corporation, Bedford, MA) and resuspended in filtered PBS for analysis by flow cytometry (CytoFlex A, Beckman Coulter).

### Measurements of perfusion pressure in the isolated perfused rat kidney

We evaluated the effect of CBF and angiotensin-II (each 100 nmol L^−1^) on vascular tone in an isolated perfused rat kidney with a constant flow of 8 mL min^–1^, as described in, e.g. [37]. Briefly, we continuously monitored perfusion pressure by a transducer (Statham P23 GB, Siemens) connected to a bridge amplifier (Hugo Sachs). We excised and immediately mounted the kidney into the perfusion system. The perfusion procedure generally followed the description given by van der Giet et al. [73]. Briefly, we perfused the isolated rat kidney by a peristaltic pump in a single-pass system with a solution containing 115 mmol L^–1^ NaCl, 4.6 mmol L^–1^ KCl, 1 mmol L^–1^ CaCl_2_, 1.2 mmol L^–1^ MgSO_4_, 1.2 mmol L^–1^ NaH_2_PO_4_, 22 mmol L^–1^ NaHCO_3_, 49 mmol L^–1^ glucose and 35 g of gelatine L^–1^ (Behringwerke) and equilibrated with 95% O_2_/5% CO_2_.

### Clinical determination of plasma CBF levels in end-stage renal disease and healthy controls

The clinical study included 17 patients who suffered from stage 5 CKD (ESRD) on regular dialysis as well as additional 10 ESRD patients before and after dialysis. The control individuals (*N* = 13) did not have CKD (controls), with estimated glomerular filtration rates ≥ 60 mL min^−1^ 1.73^–1^ m^−2^ according to the Modification of Diet in Renal Disease (MDRD) Study formula. Creatinine and haemoglobin were measured using standard autoanalyser techniques. Intact parathyroid hormone was determined by immunoassay. Plasma samples were immediately separated from blood cells and frozen until all samples were collected, and data were measured side by side within one analytical setup. Brachial blood pressure and pulse wave velocity (PWV) were measured by a single trained observer after a rest in the supine position at the dominant arm as described elsewhere [3, 51]. For details see the supplementary file.

### Statistical analyses

See supplementary methods.

## Results

### Extraction and identification of the “calcification blocking factor” from adrenal glands

**Fig. 1:**
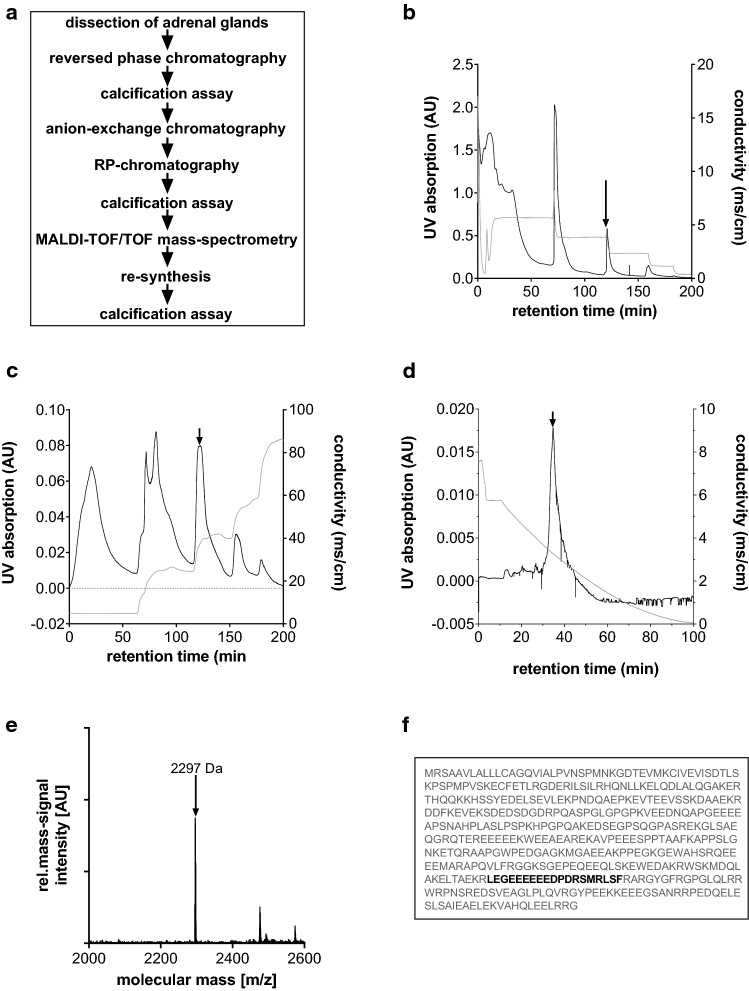
Identification and characterization of “calcification blocking factor” (CBF) from bovine adrenal glands. **a** Outline of experimental workflow for the identification and characterization of CBF, isolated from bovine adrenal glands. **b** Stepwise preparative reversed-phase chromatography of the bovine adrenal extracts (chromatographic conditions: column: Lichroprep RP C 18 (Merck); eluent A: 0.1% trifluoroacetic acid in water; eluent B: 80% acetonitrile in water; gradient: 20, 40, 60, 80, and 100% eluent B; UV absorption 280 nm). The fraction labelled by an arrow caused an inhibitory effect on vascular calcification processes of thoracic aortic rings. **c** Stepwise preparative anion-exchange chromatography of the fraction labelled by an arrow in suppl. Figure 1a (chromatographic conditions: column: SuperformanceTM 16 (Merck); eluent A: 20 mmol L^−1^ K_2_HPO_4_ in water; eluent B: 1 mol L^−1^ NaCl in 20 mmol L^−1^ K_2_HPO_4_ in water; gradient: 20, 40, 60, 80, and 100% eluent B; UV absorption 280 nm). The fraction labelled by an arrow caused an inhibitory effect on vascular calcification processes of thoracic aortic rings. **d** Linear gradient reversed-phase chromatography of the desalted fraction labelled by an arrow in suppl. Figure 1b (chromatographic conditions: column: LiChroprep RP C 18e (Merck); eluent A: 0.1% trifluoroacetic acid in water; eluent B: 80% acetonitrile in water; gradient: 1–100% eluent B over 90 min; UV absorption 280 nm). The fraction labelled by an arrow caused an inhibitory effect on vascular calcification processes of thoracic aortic rings. **e** The fraction shown in (**d**) was analysed by MALDI-TOF/TOF mass spectrometry to identify the sequence of the inhibitory factor of calcification. The mass signal (*m*/*z*) at 2297 Da [M + H]^+^ corresponds to the peptide sequence LEGEEEEEEDPDRSMRLSF, which was named “calcification blocking factor” (CBF). **f** Sequence matching: the peptide sequence of CBF represented in black characters matches the 350–369 amino acid position of bovine chromogranin A protein represented in grey characters

To identify peptides produced by the adrenal medulla involved in vascular calcification, we fractionated bovine adrenal glands by chromatographic methods to identify peptides involved in vascular calcification **(**Fig. 1a). To this end, bovine adrenal gland extracts were desalted, concentrated, and fractionated by preparative reversed-phase chromatography (Fig. 1b). Each resultant fraction was tested for its inhibitory effect on vascular calcification using the ex vivo thoracic aortic ring calcification assay [31], and fractions with inhibitory activity were then further fractionated by anion-exchange chromatography (Fig. 1c). The anti-calcific activity was tested in the resulting fractions and those that showed the activity was further purified by reversed-phase chromatography (Fig. 1d). The resulting fractions were again tested for their inhibitory effects on vascular calcification via thoracic aortic ring calcification assay as recently described [31]. Fractions showing strong inhibitory effects (indicated by the arrow in Fig. 1d) were analysed by mass spectrometry. The mass spectrum of one of these fractions at a retention time of 34.8 min showed an intense signal peak with a molecular mass (*m*/*z*) of 2297 Da ([M + H)^+^) (Fig. 1e). The peptide in this fraction was fragmented and sequenced using matrix‐assisted laser desorption/ionization time-of-flight/time-of-flight (MALDI-TOF/TOF) mass spectrometry and identified as LEGEEEEEEDPDRSMRLSF through a Mascot search. This amino acid sequence corresponds to amino acids 350–369 of bovine chromogranin A, which is a precursor to several functional peptides including vasostatin-1, vasostatin-2, pancreastatin, catestatin and parastatin, as discussed recently in detail [28] (Fig. 1f; bold). We will refer to this peptide with inhibitory properties against vascular calcification as the “calcification blocking factor” (CBF) in the following.

To test whether CBF could also be found in humans, we analysed the plasma, since peptides released by the adrenal gland can usually be found in plasma. We followed the purification procedure shown in Fig. 1a and isolated a peptide with an m/z of 2,243 ([M + H]^+^) from human plasma (Supplementary Fig. 1a). The amino acid sequence of this peptide was identified as LEGQEEEEDNRDSSMKLSF and corresponds to an amino acid sequence of human chromogranin A (Supplementary Fig. 1b; bold). To validate the amino acid sequence, the MS/MS fragment spectrum of the synthetic peptide with the amino acid sequence LEGQEEEEDNRDSSMKLSF and the isolated peptide were compared, confirming the identities of these peptides (data not shown). These data indicate that human plasma contains the CBF peptide and confirm the molecular identity.

### Characterization of the calcification inhibitory effect of CBF in vitro and in vivo

**Fig. 2:**
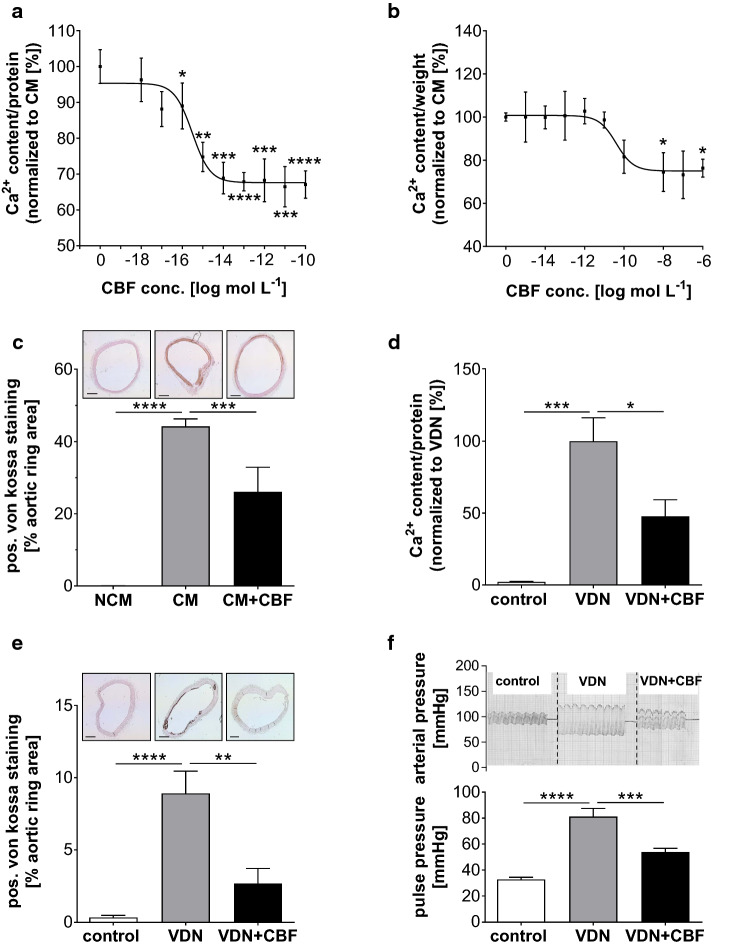
CBF inhibits calcification in thoracic aortic rings and vascular smooth muscle cells and influences pulse pressure in vivo. **a** Dose–response effect of CBF on Ca^2+^ content of cultivated human aortic smooth muscle cells. Data are shown as mean ± SEM. ***P ≤ 0.001, ****P ≤ 0.0001 compared to the calcifying condition in the absence of CBF based on one-way ANOVA. Bonferroni’s multiple comparisons were used as a post-test (*N* = 10 per group). **b** Dose–response effect of CBF on Ca^2+^ content of isolated thoracic aortic rings. Data are shown as mean ± SEM. *P < 0.05 compared to the calcifying condition in the absence of CBF based on one-way ANOVA. Bonferroni’s multiple comparisons were used as a post-test (*N* = 3–9 per group). **c** Quantification of von Kossa-stained thoracic aortic rings (original magnification × 40, scale bar 1000 μm), incubated in non-calcifying conditions (NCM; white bar) and under calcifying conditions in the absence (CM; grey bar) or presence of CBF (100 nmol L^−1^) (CM + CBF; black bar), respectively. Bars represent the mean ± SEM of the percentage of the stained area. ***P ≤ 0.001, ****P ≤ 0.0001 compared to the calcifying condition in the absence of CBF based on one-way ANOVA. Bonferroni’s multiple comparisons were used as a post-test (*N* = 9 per group). **d** Calcium content was measured in the aorta of untreated control Wistar rats (control: white bar), and VDN rats receiving a vehicle infused (VDN; grey bar) or CBF infused (31 µg kg^−1^ per day for 4 weeks) (VDN + CBF; black bars) via an osmotic pump. Data are shown as mean ± SEM. *P < 0.05, ***P ≤ 0.0001 compared with VDN group based on one-way ANOVA. Bonferroni’s multiple comparisons were used as a post-test (*N* = 6 per group). **e** Quantification of von Kossa-stained thoracic aortic rings (original magnification × 40, scale bar 1000 μm) of rats from each experimental group are shown. Bars represent the mean ± SEM of the percentage of the stained area. **P ≤ 0.001, ****P ≤ 0.00001 compared with the VDN group based on one-way ANOVA. Bonferroni’s multiple comparisons were used as a post-test (*N* = 10 per group). **f** CBF prominently prevented the VDN-induced rise in pulse pressure. Representative graphs of carotid arterial pressure recorded in a rat from each experimental group are shown. The graph represents the mean ± SEM of pulse pressure values. Pulse pressure was increased twofold in VDN rats. CBF counteracted the changes in both systolic and diastolic pressure associated with VDN and reduced the pulse pressure by 63%. ***P ≤ 0.0001; ****P ≤ 0.00001 compared with VDN group based on one-way ANOVA. Bonferroni’s multiple comparisons were used as a post-test (*N* = 10 per group)

After isolating CBF from human plasma, we characterized its effects on vascular calcification in vitro and ex vivo. To this end, a synthetic human CBF was generated and its effects on the calcification of human aortic smooth muscle cells (hAoSMCs) in vitro were investigated. The cells were cultured in the presence of high glucose to ensure stable growth. A potential effect of glucose on vascular calcification [10, 45] was eliminated by normalizing the data to the calcifying medium (CM) condition. The threshold concentration at which CBF decreased calcification in hAoSMCs was in the range of 1 amol L^−1^, and the corresponding EC_50_ was in the range of 10 amol L^−1^ (Fig. 2a). The calcium content was normalized to the protein content of the hAoSMCs following [31]. Then, we validated the effects of human CBF on the calcification of rat thoracic aortic rings ex vivo by culturing aortic rings in calcifying medium (CM) in the presence of CBF at increasing concentrations. Calcification was reduced by CBF in a dose-dependent manner, as shown by quantification of the calcium content (Fig. 2b). The calcium content was normalized to the weight of the cultivated aortic rings to avoid distorting the degree of calcification by loss of protein while releasing the proteins from the aortic rings. The threshold concentration at which CBF decreased calcification was in the order of 0.01 nmol L^−1^, and the corresponding EC_50_ value was in the order of 100 pmol L^−1^. Furthermore, reduced aortic ring calcification in the presence of CBF was confirmed by von Kossa staining, which revealed a 40% reduction in the calcified area upon treatment with CBF at 100 nmol L^−1^ compared to calcification of aortic rings cultured in CM alone (Fig. 2c).

Next, the calcification model of animals treated with vitamin D and nicotine (VDN animal) was used to assess the impact of CBF on calcification in vivo.

The calcium content of the vessels was dramatically increased in the VDN animal group receiving a vehicle infused via an osmotic pump compared to the control group (*N* = 6 each) without any treatment (Fig. 2d), but the VDN treatment did not affect the plasma concentration of murine CBF compared to the controls (0.47 ± 0.10 nmol L^−1^ vs. 0.46 ± 0.12 nmol L^−1^ (intensity of murine CBF mass signal; ns). Continuous CBF treatment via an osmotic pump for 4 weeks resulted in a plasma concentration of 5.27 ± 0.76 nmol L^−1^ without any impact on the animal morbidity or mortality, but significantly reduced the calcium content in the vessels of CBF-treated VDN rats compared to untreated VDN rats (Fig. 2d). Interestingly, using CT scan to perform the effect of CBF on bone mineralization showed a significantly lower bone density in VDN rats in comparison to the control rats (4756.98 ± 75.9 HU vs. 5328.63 ± 53.7 HU (HU: Hounsfield units; *N* = 6)). VDN rats treated with CBF show improved bone density as compared to VDN rats (5010.87 ± 62.1 HU vs. 4756.98 ± 75.9 HU; *N* = 6). In addition, the cultivation of cementoblasts for 9 days in the calcifying medium (CM) led to an increase in the calcium content in these cells in comparison to the non-calcifying condition (100.0 ± 0.0% vs 7.5 ± 5.1%; *N* = 6). However, the addition of CBF has no significant effect on the cementoblasts calcification (100.0 ± 10.4% vs 94.3 ± 23.6%; *N* = 6).

Aortic calcification was visualized by von Kossa staining of the thoracic aortic segments from control, untreated, and CBF-treated male VDN rats (*N* = 10 each). CBF treatment significantly decreased the calcified surface area by 70% (Fig. 2e). In addition, treatment with CBF in vivo decreased arterial pressure in the VDN animals and pulse pressure decreased from 81 ± 6 to 53 ± 3 mmHg (*P* ≤ 0.001) (Fig. 2f). CBF did not change the perfusion pressure of an isolated perfused kidney (33.0 ± 2.4 mmHg vs. 32.8 ± 1.7 mmHg; ns (*P* > 0.05)) nor did it change the blood pressure in non-CKD animals (SAP CBF vs control: 141.0 ± 5.1 mmHg vs. 135.2 ± 6.6 mm Hg; DAP: 108.0 ± 4.8 mmHg vs 102.4 ± 5.1 mmHg; *N* = 10 each). These data confirmed the inhibitory effects of CBF against vascular calcification in vivo in the VDN animal model through measurement of the vessel calcium content, von Kossa staining, and measurement of arterial and pulse pressure. Furthermore, the results show that CBF does not affect blood pressure directly. Only if vascular calcification has been initiated, as in VDN animals, does CBF lower blood pressure.

### Identification of the active sequence for the inhibitory effect of CBF

**Fig. 3:**
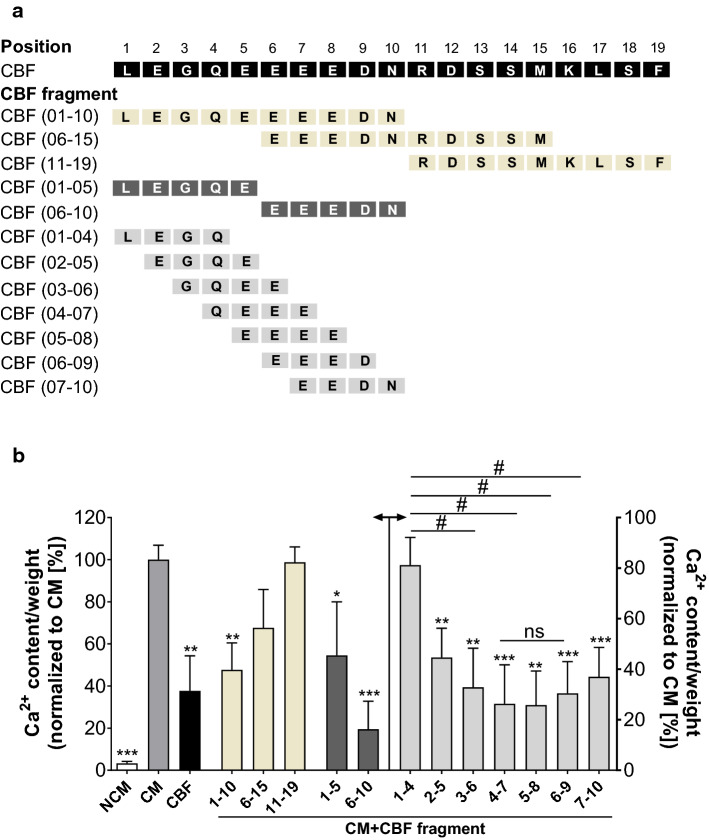
Identification of relevant amino acid sequence of CBF causing the calcification inhibitory effect. **a** Overview of the amino acid sequence of CBF fragments used for identification of the active site of CBF causing the calcification inhibitory effect. Black: amino acid sequence of CBF. Beige: CBF fragments with an amino acid sequence of 9–10 amino acids. Dark grey: CBF fragments with an amino acid sequence of five amino acids. Light grey: CBF fragments with an amino acid sequence of four amino acids. **b** Quantification of Ca^2+^ content of cultivated rat thoracic aortic rings incubated in non-calcifying conditions (NCM; white bars) and under calcifying conditions in the absence (CM; grey bar) or presence of CBF (CM + CBF; black bar) or CBF fragments (CM + CBF; beige, dark and light grey bars) as indicated in (**a**) (100 nmol L^−1^). Data are shown as mean ± SEM. **P ≤ 0.001 compared with the CM group based on one-way ANOVA. #*P* < 0.05 demonstrates significant differences in Ca^2+^ contents caused by CBF fragments with an amino acid sequence of four amino acids based on one-way ANOVA. Bonferroni’s multiple comparisons were used as a post-test (*N* = 6–9 per group). *ns* no significant difference

To identify the active sequence of CBF, we synthesized the fragments of the CBF peptide (Fig. 3a) and analysed the inhibitory effect of these peptide fragments on calcification ex vivo using thoracic aortic rings incubated in CM in the absence or presence of the CBF fragments for 7 days. Initially, the amino acid sequence of CBF was divided into two peptides consisting of ten amino acids each (CBF 1–10, CBF 6–15) and one peptide consisting of nine amino acids (CBF 11–19) (Fig. 3a, beige). CBF 01–10 significantly decreased the calcium content of the aortic rings (Fig. 3b) grown in CM in comparison to that in untreated aortic rings, while the fragments CBF 6–15 and CBF 11–19 had no significant effect. The inhibitory effects of CBF 1–10 and the complete CBF peptide (CBF 1–19) were not significantly different. Next, the inhibitory effects of the CBF 01–10 sub-fragments CBF 01–05 and CBF 06–10 (Fig. 3a; dark grey) were analysed. Both sub-fragments significantly reduced calcification in the aortic rings, although the effect was stronger in aortic rings incubated with the fragment CBF 6–10 (Fig. 3b). To further narrow down the active sequence of CBF, we analysed the fragments CBF 1–4, CBF 2–5, CBF 3–6, CBF 4–7, CBF 5–8, CBF 6–9 and CBF 7–10 (Fig. 3a; light grey). Each fragment showed an inhibitory effect on vascular calcification in the aortic rings, with the strongest inhibition caused by CBF 4–7, CBF 5–8, and CBF 6–9 (Fig. 3b). The identification of the active site of the CBF peptide has the potential to promote the development of new pharmaceuticals based on this amino acid sequence.

### Clarification of the enzymatic cleavage of CBF from chromogranin A

**Fig. 4:**
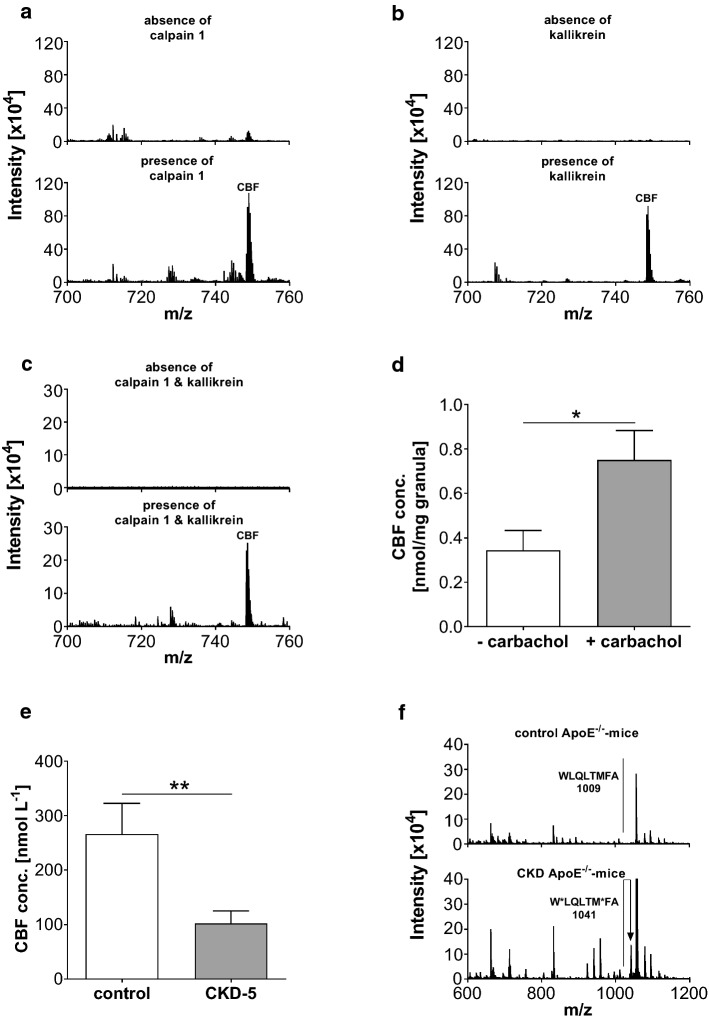
Identification of enzymes cleaving CBF from chromogranin A. **a** LC-qTOF mass spectrometric analyses of LAKELTAEKR-LEGQEEEEDNRDSSMKLSF incubated in the absence (upper panel) and presence (lower panel) of kallikrein. The molecular mass (*m*/*z*) of 748.66 [M + 3H]^3+^ of the average spectrum at retention time range 18.8–19.7 corresponds to CBF. **b** LC-qTOF mass-spectrometric analyses of LEGQEEEEDNRDSSMKLSF-RARAYG FRGP incubated in the absence (upper panel) and presence (lower panel) of calpain 1. The molecular mass (*m*/*z*) of 748.66 [M + 3H]^3+^ of the average spectrum at retention time range 18.8–19.7 corresponds to CBF. **c** LC-qTOF mass-spectrometric analyses of chromogranin A incubated in the absence (upper panel) and presence (lower panel) with both kallikrein and calpain 1. The molecular mass (*m*/*z*) of 748.66 [M + 3H]^3+^ of the average spectrum of retention time range 18.8–19.7 corresponds to CBF. **d** Quantification of CBF in the supernatant of granules from bovine adrenal glands unstimulated and stimulated with carbachol (0.1 mmol L^−1^). The release of CBF from granula of adrenal glands into the culture media was doubled after stimulation. Data are shown as mean ± SEM. *P < 0.05 compared with the group without carbachol treatment based on unpaired *t* tests (*N* = 5 in each group). **e** The concentration of CBF in plasma of healthy controls (*N* = 13) and CKD-5 patients (*N* = 17) is reported in nmol L^−1^. CBF concentrations were decreased in patients suffering from CKD-5. ** indicates *P* ≤ 0.001compared to healthy based on unpaired *t* tests. **f** Characteristic MALDI mass spectra of the chromogranin A cleaving enzyme calpain 1 isolated from control C57BL/6J ApoE^−/−^ mice (upper panel) and CKD adenine C57BL/6J ApoE^−/−^ mice (lower panel) demonstrating CKD-associated enzyme modification by oxidation. The induction of CKD was performed by a 1.5-week diet of 0.3% adenine. A stable CKD condition was subsequently achieved with 0.15% adenine for 4.5 weeks. In addition, the animal received a high-fat diet during the whole time of experiments starting 4 weeks before the induction phase (*N* = 5 per group)

As CBF was identified as a peptide derived from the protein chromogranin A, we next investigated the enzymes that cleave CBF from chromogranin A using the Proteasix database. For these experiments, elongated CBF peptides with ten additional amino acids of chromogranin A at either the N- or the C-terminus were used. First, we examined whether calpain 1 cleaves the N-terminus of CBF from chromogranin A, by incubating LAKELTAEKR-CBF with and without calpain 1 for 48 h. While incubation of LAKELTAEKR-CBF in the absence of enzymes caused no molecular mass signal for CBF (Fig. 4a), an intense mass signal at 748.66 ([M + 3H]^3+^) was detected after incubation in the presence of calpain 1, demonstrating the N-terminal cleavage of CBF from LAKELTAEKR-CBF (Fig. 4a; lower spectrum). Next, we studied whether kallikrein cleaves the C-terminus of CBF from chromogranin A, by incubating CBF-RARAYGFRGP in the presence and absence of kallikrein for 48 h. While incubation of CBF-RARAYGFRGP in the absence of enzymes caused no molecular mass signal for CBF (Fig. 4b; upper spectrum), an intense mass signal at 748.66 ([M + 3H]^3+^) was detected after incubation in the presence of kallikrein, demonstrating the C-terminal cleavage of CBF from CBF-RARAYGFRGP (Fig. 4b; lower spectrum). Incubation of chromogranin A in the presence of both calpain 1 and kallikrein, but not in their absence, caused an intense mass signal at 748.66 ([M + 3H]^3+^), demonstrating the cleavage of CBF from chromogranin A (Fig. 4c; lower spectrum).

After identification of the enzymes cleaving CBF from chromogranin A, the stimulus that induces CBF release from the adrenal glands by a physiological stimulus was studied. Carbachol, which binds and activates acetylcholine receptors, significantly increased the release of CBF from adrenal glands (Fig. 4d), suggesting that CBF release may involve acetylcholine receptors. To examine whether CBF is proteolyzed from intracellular or extracellular chromogranin A, adrenal gland chromaffin granules were isolated by density gradient centrifugation and chromatographic techniques. CBF was detected in one of the fractions from the adrenal gland chromaffin granules, suggesting that CBF is a component of the chromaffin granules of adrenal glands (Supplementary Fig. 1c).

### Quantification of CBF plasma concentration in chronic kidney disease

Next, we validated the relevance of CBF to human pathophysiology by quantifying the concentration of CBF in plasma in ESRD patients, who suffer from increased vascular calcification and healthy control subjects using mass spectrometry as described above. Table 2 shows the clinical and biochemical characteristics of ESRD patients and healthy control subjects. The CBF plasma concentration was significantly lower in ESRD patients compared to healthy control subjects (102.4 ± 23.0 nmol L^−1^ and 266.1 ± 56.4 nmol L^−1^, *N* = 17 and *N* = 13, respectively; *P* < 0.05) (Fig. 4e). These results suggest that CBF might have an impact on the increased vascular calcification rate in these patients, most likely caused by modification of the enzyme activity of calpain 1 and/or kallikrein in these patients. The molecular structure of both calpain 1 and/or kallikrein from adrenal glands of C57BL/6J ApoE^−/−^ adenine CKD mice were investigated by mass spectrometry, demonstrating post-translational modification of calpain 1 in the CKD but not in the control animals (Fig. 4f). To monitor both the induction and maintenance of CKD in the animals, plasma urea and creatinine levels were measured. An increase of plasma urea levels by 3.9 ± 0.3-fold and plasma creatinine level by 3.6 ± 0.2-fold in the CKD model compared to control animals during the induction phase was observed. While during the maintenance phase, urea and creatinine levels remained elevated, 2.4 ± 0.2-fold increase for urea and 1.7 ± 0.1-fold for creatinine, respectively, were found compared to control animals. ApoE^−/−^ is a strong pro-inflammatory and oxidative stress mutation [38, 53]; nevertheless, it most accurately represents the clinical parameters of CKD patients. However, to evaluate whether the post-translational modification of calpain 1 by ApoE^−/−^ induced oxidative stress, the experiments were repeated in wild-type mice. Again, oxidation of calpain 1 could only be observed in CKD animals, but not in control animals (Supplementary Fig. 2a). In addition, the monitoring of CKD induction in wild-type animals showed an increase of plasma urea levels by 2.4 ± 0.3-fold and plasma creatinine level by 1.6 ± 0.2-fold in the CKD model compared to control animals during the induction phase. While during the maintenance phase, urea and creatinine levels remained elevated, 1.7 ± 0.15-fold increase for urea and 1.2 ± 0.2-fold for creatinine compared to control animals.

**Table 2:** Clinical and biochemical characteristics of ESRD patients and control subjects (values are mean ± SEM. *P < 0.05, ***P ≤ 0.001 and ****P ≤ 0.0001 compared with healthy control subjects based on unpaired *t* tests)

Parameter	Healthy control subjects (*N* = 13)	ESRD patients (*N* = 17)	Significance
Age (years)	58.7 ± 3.1	52.8 ± 3.4	n.s.
Blood pressure (mmHg)			
Systolic	125.9 ± 5.6	125.0 ± 5.9	n.s.
Diastolic	70.8 ± 2.0	71.8 ± 3.9	n.s.
Weight (kg)	82.6 ± 4.9	77.0 ± 3.6	n.s.
BMI (kg m^−2^)	27.9 ± 1.2	26.0 ± 124	n.s.
Serum creatinine (mg dL^−1^)	1.04 ± 0.03	6.60 ± 0.49	***P ≤ 0.001
eGFR (MDRD (mL min^−1^ 1.73^–1^ m^−2^)	69.7 ± 2.7	9.5 ± 0.6	***P ≤ 0.001
Haemoglobin (g L^−1^)	219.2 ± 36.8	193.6 ± 8.0	*P < 0.05
PTH (pg mL^−1^)	39.7 ± 3.7	231.3 ± 37.4	****P ≤ 0.0001
Serum calcium (mg dL^−1^)	2.2 ± 0.5	2.3 ± 0.7	n.s.
Serum phosphate (mg dL^−1^)	3.3 ± 0.6	5.4 ± 0.8	***P ≤ 0.001

In vitro post-translational modification of calpain 1 results in a significant decrease of the enzymatic cleavage of CBF from the elongated CBF by 52% (0.01063 ± 0.00087 vs 0.0051 ± 0.00069; *N* = 3) (Supplementary Fig. 2b). In addition, the effect of a hemofiltration treatment on the CBF plasma concentration was investigated. A 4-h conventional hemofiltration significantly decreased the plasma CBF concentration in ESRD patients (Supplementary Table 1) (before vs after hemofiltration: 1062.0 ± 730.9 nmol L^−1^ vs. 478.4 ± 415.4 nmol L^−1^, *N* = 10, respectively; *P* < 0.05).

### Identification of the pathway mediating the inhibitory effect of CBF

**Fig. 5:**
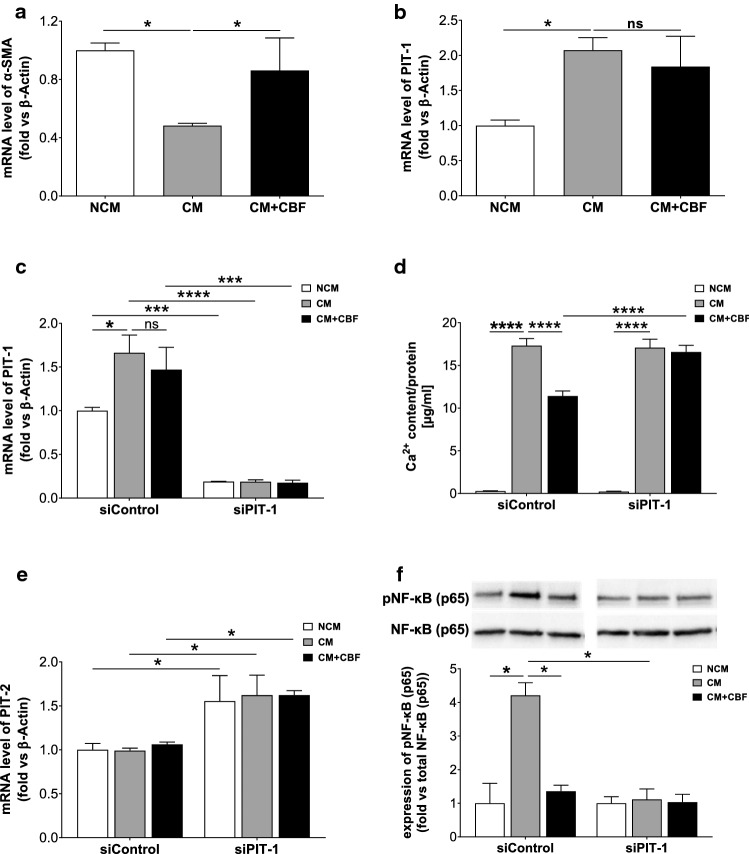
The sodium-dependent phosphate cotransporter 1 (PIT-1) mediates the inhibitory effect of CBF. **a** Relative quantification of α-smooth muscle actin protein (α-SMA) gene expression by RT-qPCR analyses. The mRNA level of α-SMA significantly decreased in the case of cultivating the cells under calcifying conditions (calcifying medium (CM)) compared to non-calcifying conditions (non-calcifying medium (NCM)), but not in the presence of CBF. Data are shown as mean ± SEM. *P < 0.05 compared with CM based on one-way ANOVA. Bonferroni’s multiple comparisons were used as a post-test (*N* = 3 per group). **b** Relative quantification of PIT-1 gene expression by RT-qPCR analyses. The increased gene expression in human aortic smooth muscle cells cultured for 7 days in calcifying medium (CM; grey bar) vs. non-calcifying medium (NCM; white bar) was not affected by CBF (100 nmol L^−1^) (black bar). Data are shown as mean ± SEM. *P < 0.05 compared with CM based on one-way ANOVA. Bonferroni’s multiple comparisons were used as a post-test (*N* = 3 per group). **c** Quantification of PIT-1 gene expression in VSMCs transfected with a control and PIT-1 siRNA, respectively, after incubation in non-calcifying medium (NCM; white bar) or calcifying medium in the absence (CM; grey bar) or presence (black bar) of CBF. Data are shown as mean ± SEM. *P < 0.05; ***P ≤ 0.001 and ****P ≤ 0.0001. The data based on one-way ANOVA. Bonferroni’s multiple comparisons were used as a post-test (*N* = 6 per group). **d** Quantification of Ca^2+^ content in transfected human aortic smooth muscle cells with 120 µM control siRNA or 120 µM PIT-1 siRNA after incubation with non-calcifying conditions (non-calcifying medium (NCM; white bar) and under calcifying conditions in the absence (CM; grey bar) or presence (black bar) of CBF (100 nmol L^−1^). Data are shown as mean ± SEM. ****P ≤ 0.0001 based on two-way ANOVA. Bonferroni’s multiple comparisons were used as a post-test (*N* = 3 per group). **e** Quantification of phosphate transporter 2 (PIT-2) gene expression in VSMCs transfected with a control and PIT-1 siRNA, respectively, after incubation in non-calcifying medium (NCM; white bar) or calcifying medium in the absence (CM; grey bar) or presence of CBF (black bar). Data are shown as mean ± SEM. *P < 0.05. The data based on one-way ANOVA. Bonferroni’s multiple comparisons were used as a post-test (*N* = 5 per group). **f** Western blot and quantitative analysis of NF-κB (p65) activation in VSMCs transfected with a control and PIT-1 siRNA, respectively, after incubation in non-calcifying medium (NCM; white bar) or calcifying medium in the absence (CM; grey bar) or presence of CBF (black bar). Data are shown as mean ± SEM. *P < 0.05. The data based on one-way ANOVA. Bonferroni’s multiple comparisons were used as a post-test (*N* = 3 per group)

To understand how CBF inhibits vascular calcification, we studied the involvement of CBF in VSMC transdifferentiation into osteoblast-like cells within the vascular wall, as this is an essential step in the pathophysiology of vascular calcification. The expression of α-smooth muscle actin (α-SMA) as the predominant actin isoform in VSMCs was quantified in hAoSMCs cultivated in non-calcifying conditions, as well as under calcifying conditions in the absence or presence of CBF. Figure 5a shows that the expression of α-SMA in cells incubated with the calcifying medium is reduced compared to the cells treated with CBF. These results suggest that the transdifferentiation of smooth muscle cells to osteoblast-like cells was blocked by CBF. In addition, calcifying conditions caused a strong and significant reduction of the cell viability compared to non-calcifying conditions; however, this effect was significantly decreased by the cultivation of VSMCs under calcifying conditions in the presence of CBF (Supplementary Fig. 3a). For validation of the effect of CBF on VSMC viability under calcifying media conditions, the effect of CBF on cell apoptosis was additionally analysed by TUNEL staining in vitro (Supplementary Fig. 3b). Again, the cultivation of VSMC under calcifying conditions in the absence of CBF caused an increased apoptosis rate of the cells. This effect was significantly decreased by the cultivation of VSMCs under calcifying conditions in the presence of CBF (Supplementary Fig. 3c). Furthermore, reduced calcification-induced cell apoptosis by CBF was shown in vivo in the medial layer of aortas of VDN rats treated with CBF (Supplementary Fig. 3d). Furthermore, the effect of CBF on the genesis of apoptotic bodies was investigated in vitro by FACS quantification of annexin V-positive apoptotic bodies released by VSMCs after cultivation in non-calcifying conditions, calcifying conditions and calcifying conditions in the presence of CBF, respectively. Thereby, an increased number of apoptotic bodies were detected in the case of cells incubated in calcifying conditions compared to cells incubated in non-calcifying conditions. The effect of calcifying conditions on the number of apoptotic bodies was reversed by the presence of CBF (Supplementary Fig. 3e).

To determine the interaction partner of CBF, potential candidates were screened and excluded, such as BMP2 receptor as a prime candidate via plasmon resonance spectrometry as well as other receptors such as TGFβ-receptor and TNFα-receptor using co-immunoprecipitation (CoIP) (Supplementary Fig. 4a and 4b). Since the type III NAPi cotransporter, PIT-1, is the predominant form of the sodium-dependent phosphate cotransporter expressed in human VSMCs [52] and it has been identified as a pivotal transporter involved in phosphate-induced VSMC transdifferentiation [16, 24], we next analysed the effect of CBF on PIT-1 expression under calcifying conditions. PIT-1 expression was significantly increased in VSMCs incubated for 5 days with CM compared to NCM-cultured cells independently of the presence or absence of CBF (Fig. 5b). To analyse whether PIT-1 silencing influences the CBF-induced anti-calcific effect, VSMCs were transfected with PIT-1 and control siRNA, respectively. The expression of PIT-1 was analysed using RT-qPCR after 5 days of incubation. Figure 5c shows an ~ 80% PIT-1 knockdown after transfection of cells with the PIT-1 siRNA compared to siControl transfected cells. In addition, neither the calcifying medium nor the CBF peptide had any effect on PIT-1 expression after silencing (Fig. 5c). However, knockdown of PIT-1 in hAoSMCs led to a significant reduction in the inhibitory effect of CBF on calcification compared to that observed in cells transfected with control siRNA, where a 35% inhibition of calcium content by CBF treatment was observed (Fig. 5d). These data demonstrated that CBF acts on the activity of PIT-1 receptor as a result of the binding to the transporter, but not on receptor expression. Since an increased calcification was observed in the CM cultured cells despite the silencing of PIT-1 (Fig. 5d, grey bars), the expression of PIT-2 in PIT-1 silenced cells was analysed to clarify whether the calcification is mediated by type III NaPi cotransporter PIT-2. As shown in Fig. 5e, an increase of PIT-2 expression was detected after PIT-1 silencing compared to control siRNA transfected cells, demonstrating PIT-2 adopts the function of PIT-1, which not is affected by CBF. Since recent data suggest a relationship between PIT-1 and NF-κB [42] with high phosphate concentrations enhancing vascular calcification processes by activating NF-κB in VSMCs [82], next the effect of CBF on activation of NF-κB in VSMCs was investigated. While an increase in NF-κB activation was observed in cells cultivated in CM conditions compared to NCM-cultured cells (3.4 ± 0.3 vs 1.0 ± 0.4; *N* = 5), the NF-κB activation in VSMCs was significantly reduced in the presence of CBF (3.4 ± 0.3 vs 0.9 ± 0.2; *N* = 5) (Supplementary Fig. 4c). These data were confirmed in cells transfected with the control siRNA, while after PIT-1 silencing the NF-κB activation was significantly reduced in cells cultivated in calcifying conditions (Fig. 5f). The intracellular phosphate amount of VSMC after cultivation of VSMCs in non-calcifying conditions, calcifying conditions and calcifying conditions in the presence of CBF, respectively, was quantified. A significantly increased concentration of intracellular phosphate was detected under calcifying conditions in comparison to non-calcifying conditions (100 ± 5.2%vs 63.9 ± 6.2%; *N* = 5). This effect was counteracted in the presence of CBF confirming the inhibitory effect of CBF on the calcification processes (100 ± 5.2% vs 83.4 ± 2.4%; *N* = 5) (Supplementary Fig. 4d).

**Fig. 6:**
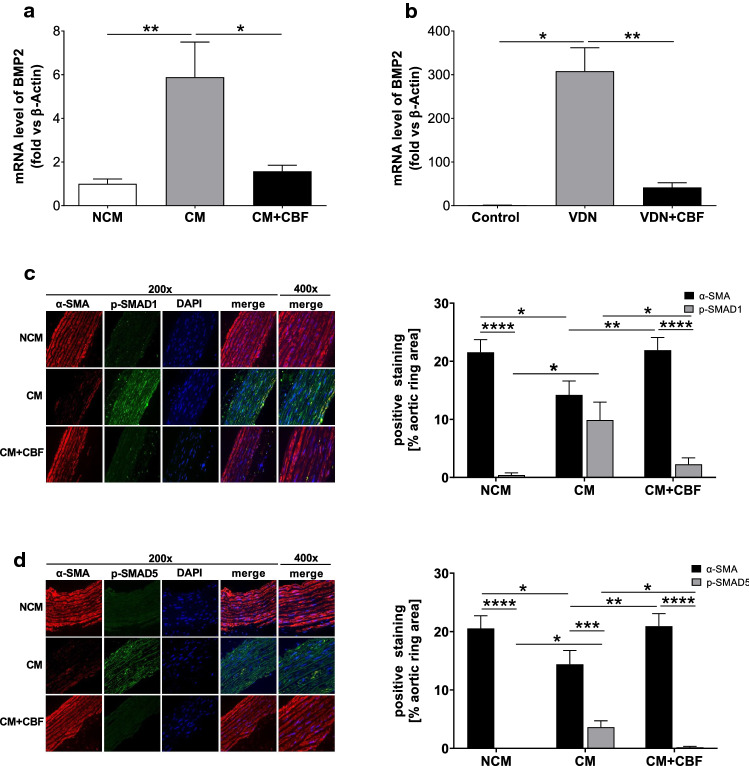
CBF inhibits the BMP2/p-SMAD pathway. **a** Relative quantification of bone morphogenetic protein 2 (BMP2) gene expression in vitro by RT-qPCR analyses. The increased gene expression in human aortic smooth muscle cells cultured for 7 days in calcifying medium ((CM); grey bar) vs. non-calcifying medium ((NCM); white bar) was diminished in the presence of CBF (100 nmol L^−1^) (black bar). Data are shown as mean ± SEM. *P < 0.05; **P ≤ 0.01 compared with CM based on one-way ANOVA. Bonferroni’s multiple comparisons were used as a post-test (*N* = 6 per group). **b** Relative quantification of bone morphogenetic protein 2 (BMP2) gene expression in vivo by RT-qPCR analyses. The increased gene expression of the aorta of VDN rats (grey bar) vs. control rats (white bar) was limited in rats treated with CBF (31 µg kg^−1^ per day for 4 weeks) (black bar). Data are shown as mean ± SEM. *P < 0.05; **P ≤ 0.01 compared with VDN group based on one-way ANOVA Bonferroni’s multiple comparisons were used as a post-test (*N* = 5 per group). **c**, **d** Relative quantification of (**c**) α-smooth muscle actin (α-SMA) and phospho-SMAD1 (p-SMAD1) and **d** α-SMA and phospho-SMAD5 (p-SMAD5) of rat thoracic aortic rings cultivated in non-calcifying medium (NCM; white bar) and calcifying medium in the absence (CM; grey bar) or presence of CBF (100 nmol L^−1^) (black bar). Representative images (original magnification × 200, Scale bar 200 μm) of **c** α-SMA (red) and p-SMAD1 or **d** α-SMA (red) and p-SMAD5 (green), DAPI staining of nuclei (blue), and the corresponding merged images are shown. Data are shown as mean ± SEM. *P < 0.05; **P ≤ 0.01; ***P ≤ 0.0001, ****P ≤ 0.00 = 01. The data based on one-way ANOVA. Bonferroni’s multiple comparisons were used as a post-test (*N* = 6–9 in each group)

Since phosphate uptake via PIT-1, as well as activation of NF-κB, promotes the expression of BMP2 in VSMCs [19, 58, 83] and the BMP2/p-SMAD pathway is strongly involved in vascular calcification [46, 77], we next analysed whether BMP2 expression in vitro and in vivo is modified by CBF. Calcifying conditions caused a strong and significant increase in BMP2 mRNA expression compared to that in cells cultured under non-calcifying conditions, an effect that was reversed when the cells were incubated in the presence of CBF (Fig. 6a). This effect was confirmed in vivo by analysing aortas isolated from control, VDN, and VDN rats continuously treated with CBF (Fig. 6b). In addition, the knockdown of PIT-1 in VSMCs led to the significant reduction of BMP2 expression in cells cultured in calcifying conditions (Supplementary Fig. 4e).

Next, we analysed the phosphorylation levels of the downstream components of the BMP2 pathway SMAD1 and SMAD5 as well as α-SMA expression, as a predominant actin isoform in VSMCs, in thoracic aortic rings cultured under non-calcifying conditions and in CM in the absence and presence of CBF using immunofluorescence microscopy. The α-SMA expression was significantly reduced by the calcifying medium in comparison to non-calcifying conditions. This effect was reversed in the presence of CBF (Fig. 6c, d). In contrast, SMAD1 and SMAD5 phosphorylation significantly increased in aortic rings incubated under calcifying medium and the signal was effectively inhibited by CBF (Fig. 6c, d). Similar results were obtained when the expression of α-SMA and phosphorylation of SMAD1 and SMAD5 was quantified in aortas isolated from control rats or VDN rats with or without treatment via continuous infusion of CBF (Supplementary Figs. 4f and 4 g). These results indicate that CBF treatment inhibits SMAD1 and SMAD5 activation under calcifying conditions [2, 47].

**Fig. 7:**
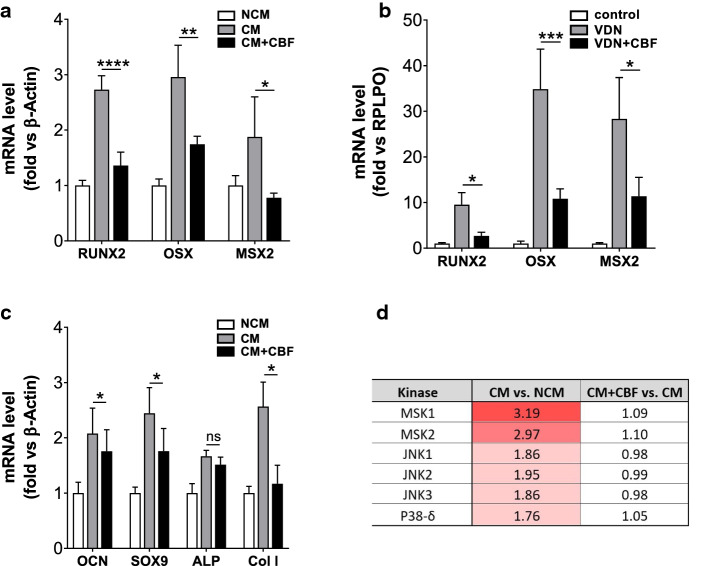
CBF reduces the expression of genes involved in osteoblast differentiation. **a**, **b** Relative quantification of runt-related transcription factor 2 (RUNX2), osterix (OSX) and msh homeobox 2 (MSX2) gene expression in (**a**) in vitro using hAoSMCs and (**b**) in vivo, respectively, by RT-qPCR analyses. Data are shown as mean ± SEM. *P < 0.05, **P ≤ 0.01, ***P ≤ 0.001, ****P ≤ 0.0001 compared with CM based on one-way ANOVA. Bonferroni’s multiple comparisons were used as a post-test (*N* = 4–6 in each group). **c** Relative quantification of osteocalcin (OCN), SOX9, alkaline phosphatase (ALP) and type 1 collagen (Col l) gene expression in vitro using hAoSMCs by RT-qPCR analyses. Data are shown as mean ± SEM. *P < 0.05 compared with CM based on one-way ANOVA. Bonferroni’s multiple comparisons were used as a post-test (*N* = 5 in each group). **d** Based on kinase activity profiling, kinase ranking scores (based on their significance and specificity in terms of the set of peptides used for the corresponding kinase) are shown for BMP-2-related kinases (*N* = 3 per condition)

Since the change from the contractile VSMCs to an osteo/chondrogenic phenotype is characterized by gain of osteochondrogenic markers [18], we assessed the effect of CBF on the relative mRNA expression of different genes including runt-related transcription factor 2 (RUNX2), a key transcription factor associated with osteoblast differentiation and upregulated in response to pro-calcification. Next, the effect of CBF on the expression of two other genes encoding pro-calcification transcription factors, msh homeobox 2 (MSX2) and osterix (OSX) in vitro using hAoSMCs as well as in vivo was investigated. Calcifying conditions caused an increase in the mRNA expression of the transcription factors compared to non-calcifying conditions (Fig. 7a); CBF reversed this effect (Fig. 7a). This effect was confirmed in vivo analysing aortas isolated from control, VDN rats, and VDN rats continuously treated with CBF (Fig. 7b). To characterize the osteogenic phenotype in the presence of CBF in more detail, we analysed the downstream genes activated by these transcription factors, regulating vascular calcification including osteocalcin, SOX9, alkaline phosphatase (ALP) and type 1 collagen. The gene expressions were investigated in vitro in HAoSMCs incubated in non-calcifying (NCM) and calcifying (CM) conditions in the absence and the presence of CBF. An increased expression was detected for osteocalcin, SOX9 and type 1 collagen, which was significantly reduced in the presence of CBF. No significant differences were detected for ALP (Fig. 7c).

Together, these results demonstrate that osteogenic genes are regulated by BMP2. In addition to BMP2/p-SMAD signalling, MAPK cascades represent an alternative, non-canonical pathway for BMP2 signal transduction [9, 33] to induce osteogenic gene expression and influence calcification [79, 84]. In line with the BMP2 expression, kinome analysis revealed that calcifying conditions caused a strong increase in p38 and JNK activity, together with its downstream targets MSK1 and MSK2, respectively (Fig. 7d; [65]), compared to cell culture in non-calcifying conditions. Importantly, the presence of CBF almost completely abolished this effect of the calcification medium. As p38 and JNK can promote the expression and activation of osteogenic genes, like RUNX2 [5], this further confirms the importance of BMP2 signalling in the inhibitory role of CBF on calcification.

## Discussion

Imbalances between inhibitory and inducing mediators are known to be involved in vascular calcification [39]. Given the wealth of peptide hormones synthesized by the adrenal medulla and their involvement in vascular calcification [81], we hypothesized that unknown adrenal-derived mediators regulate osteoblastic transdifferentiation, the main feature of vascular calcification. We identified a novel peptide CBF as well as a specific role for the adrenal glands in vascular calcification in humans. CBF is derived from the protein chromogranin A, which is expressed in the secretory cells of different tissues of neuronal, neuro-endocrine, and endocrine origin [27]. This protein, initially identified as a tumour marker [20], is associated with CV (patho)physiology [72]. Chromogranin A is a precursor of several biologically active peptides, e.g. vasostatin-1 (human CHGA) [57, 60, 69], vasostatin-2 (human CHGA 1–113) [8, 69], vasoconstriction inhibiting factor (VIF) [61], and catestatin (human CHGA 352–372) [60, 62], all of which are essential for the genesis and/or progression of CV disease. For example, in hypertension, the plasma concentration of chromogranin A is increased, while the catestatin plasma concentration is decreased [76]. The six N-terminal amino acids of CBF overlap with the C-terminal amino acids of catestatin (also named LS21 [49]); however, this short amino sequence is not relevant for the inhibitory effects of CBF as shown in Fig. 3. CBF is thus the first identified, very potent calcification inhibitor secreted by adrenal glands.

The incubation of elongated CBF peptides with calpain 1 and kallikrein demonstrates that CBF can be cleaved from chromogranin A. Disturbed activities of these enzymes might be a mechanism by which CBF plasma levels might be altered, which would ultimately affect the inhibitory function of CBF on vascular calcification. For instance, the CBF-cleavage enzymes could be inhibited by uremic toxins [64] and post-translational modifications [21] in patients with CKD. This would reduce the plasma concentration of CBF in these patients. These mechanisms may offer new options to treat or prevent vascular calcification since we show that CBF affects calcification processes in vitro and ex vivo.

The in vivo relevance of CBF in vascular calcification was demonstrated using a preclinical animal model of elastocalcinosis caused by the deposition of hydroxyapatite on the elastic lamellae of the vessel. Since the inhibitory effect of CBF in vivo is significantly stronger than in vitro and ex vitro, respectively, in vitro and ex vivo approaches are suitable for characterizing CBF, but in vivo studies are essential for determining its potency.

Interestingly, CBF not only reduced the vascular calcium content but also reduced pulse pressure in vivo, a reliable parameter for [22, 23] and established consequence of arterial stiffness in calcified vessels [4]. A direct effect of CBF on blood pressure could be excluded based on the in vitro and ex vivo as well as the in vivo experiments using non-CKD animals.

For clinical applications, knowledge of the active sequence of CBF is essential. The active sequence of CBF contains one of the amino acids glutamine, glycine and aspartic acid and five glutamic acid residues. Investigating the inhibitory effect of shorter peptide fragments of CBF with four amino acids shows that several of the peptides with four amino acids have comparable effects. The comparable effect of these peptides can be ascribed to the comparable chemical composition of the peptides. The inhibitory effect is likely caused by the glutamic acid residues of the short peptides. The presence of a carboxylic acid next to a primary amide or two carboxylic acids might be relevant for the inhibitory effect of CBF and/or its fragments. Direct binding of calcium to the amino acid motif might not be the primary effect of CBF, as the calcium concentration exceeded the CBF concentration in the medium. As such, direct binding would not significantly affect the calcium concentration. The identification of the active sequence of CBF is highly relevant to the development of peptide mimetics for future therapeutic approaches.

To evaluate the relevance of CBF in humans, the plasma levels of CBF in healthy control subjects and ESRD patients suffering from increased vascular calcification were quantified in two separate small cohorts. Since no appropriate antibody for CBF is available, mass spectrometry-based approaches were used for the quantification of both cohorts. The CBF concentrations found in plasma from healthy control subjects were shown to be effective in both our in vitro and ex vivo experiments. While the plasma concentration of CBF is higher than the EC_50_ value of CBF determined by the dose–response curve, the tissue levels of CBF are likely lower than the plasma level owing to several factors, such as the endothelial barrier to CBF, enzymatic degradation, binding, or other effects. The lower serum concentration of CBF observed in patients with ESRD compared to healthy control subjects suggests that the CBF deficit in these patients might account, at least in part, for the increased vascular calcification commonly observed in these patients. Moreover, the quantification of CBF levels in ESRD patients undergoing dialysis within the second cohort showed that dialysis might also contribute to diminished CBF levels. Since chromogranin A levels are markedly increased in renal failure [43, 48], it seems very unlikely that decreased CBF levels are the consequence of decreased precursors. Either an increased degradation or a diminished synthesis might contribute to the alterations of CBF plasma concentration in renal failure. A direct comparison of the mean values of the two studies is only viable to a limited extent of limited potential due to the limited number of persons included. Furthermore, the samples in question were taken independently of each other and with different analytical devices, which may lead to deviations in the respective measured values between different cohorts if the methods are not validated in interlaboratory comparisons [74]. Due to the high pathophysiological relevance of CBF, demonstrated in this study, the clinical effect of CBF has to be further validated in larger cohorts in multicentric studies. As CKD-associated post-translational modification frequently occur under the uremic burden and increased plasma concentration of reactive metabolic by-products, leading to the functional impairment of protein and enzymes, the CBF cleaving enzymes from chromogranin A were screened using mass spectrometry for post-translational modifications. Indeed, the post-translational modification by calpain 1 resulted in a reduction of enzymatic activity by 52% and, at least in part, might explain the reduced CBF levels in CKD patients (Supplementary Fig. 2b). The impact of CBF of vascular calcification in the human situation has to be validated in larger clinical studies with an increased number of patients to determine whether CBF is usable as a preventive agent before the onset of vascular calcification as well as its application as a therapeutic and interventive compound to inhibit ongoing or even reverse existing calcification.

Since the concentrations of calcium and phosphate are magnitudes higher compared to the concentration of CBF, the inhibitory effect of CBF is not mainly caused by direct interactions between CBF and calcium/phosphate. In our investigation to determine the interaction partner of CBF, a list of potential candidates was screened and excluded, such as BMP2 receptor as a prime candidate as well as other receptors such as TNFα-receptor and TGFβ-receptor. The transdifferentiation of VSMCs to cells to an osteoblast-like phenotype is essential for the pathophysiology of vascular calcification. CBF blocked VSMC transdifferentiation, as demonstrated by the increased expression of the VSMCs differentiation marker α-SMA in vitro, in vivo and ex vivo. In addition, knockdown in hAoSMCs of the type III NaPi cotransporter PIT-1, involved in phosphate-induced VSMC transdifferentiation [16], led to a reduction in the inhibitory effect of CBF on vascular calcification in these cells. However, although PIT-1 expression is induced by calcifying medium and decreased using siRNA, there is no effect of PIT-1 silencing on calcification in VSMCs. Nevertheless, a significant reduction in the inhibitory effect of CBF on calcification was observed compared to cells transfected with control siRNA. This effect is to be explained by the increased PIT-2 expression induced by the knockdown of PIT-1 as described in [6, 7, 16]. These results demonstrate the essential function of PIT-1 in the inhibition of vascular calcification by CBF.

Furthermore, a high phosphate concentration enhances vascular calcification processes by activating NF-κB in VSMCs [82] and downstream activation of BMP2 expression [19, 83]. Recent studies suggest that this effect is likely mediated by the interaction of PIT-1 and NF-κB [42]. This is in accordance with our study, which showed that incubation of VSMC in calcifying medium caused increased activation of NF-κB. The presence of CBF in the calcifying medium significantly reduced this activation of NF-κB.

**Fig. 8:**
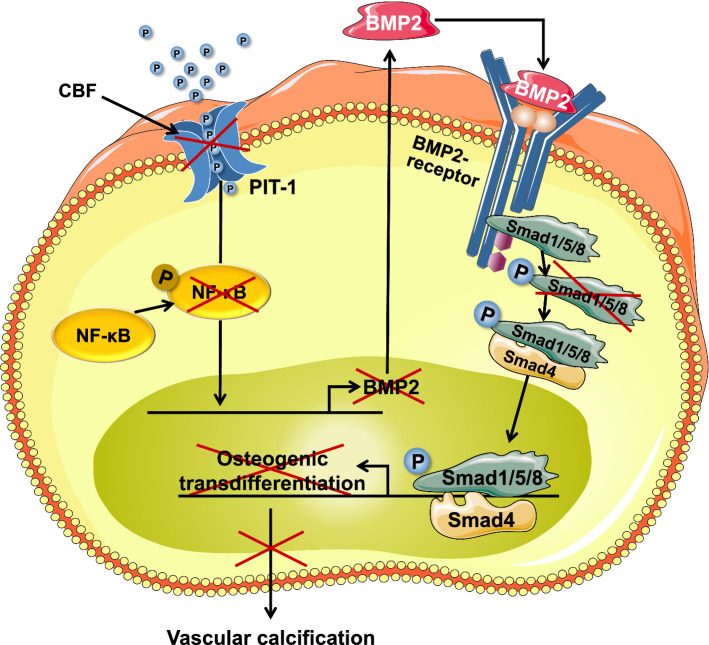
Effect of CBF on vascular calcification processes is mediated by the PIT-1/NF-κB/BMP2/p-SMAD pathway. CBF affects the transport of phosphates by the type III NaPi cotransporter PIT-1. CBF inhibits the phosphorylation and subsequent activation of NF-κB (p65), decreased the BMP2 expression and inhibits the BMP2 pathway via inhibition of SMAD1/5/8 phosphorylation, the associated expression of osteogenic transdifferentiation genes and ultimately the vascular calcification of VSMCs

The BMP2/p-SMAD pathway is involved in VSMC transdifferentiation to cells with an osteoblastic phenotype, which is associated with enhanced vascular calcification [32]. The expression of BMP2 was decreased in vitro as well as in vivo by chronic administration of CBF. In addition, CBF treatment counteracted increases in the levels of p-SMAD1 and p-SMAD5 in thoracic aortic rings in culture as well as in aortas from VDN rats. Phosphorylation of SMAD1 and SMAD5 leads to their association with co-SMAD4. This complex translocates to the nucleus, where it regulates the expression of osteogenic transdifferentiation genes, including RUNX2, MSX2 and OSX [32]. Overall, these results show that CBF decreases arterial calcification through prevention of VSMC transdifferentiation into osteoblast-like cells within the vascular wall via PIT-1 and by inhibition of NF-κB inactivation and the subsequent BMP2/p-SMAD pathway (Fig. 8).

In addition to osteogenic transdifferentiation, increased apoptosis, as well as a reduced proliferation of VSMCs, have been suggested as a highly relevant mechanism of cardiovascular calcification [44, 66]. Cellular fragmentation caused by apoptosis results in the formation of apoptotic bodies containing alkaline phosphatase and NTP pyrophosphohydrolase, which leads to calcium precipitation [26] as an initial step and ultimately to calcified vessels [55]. Our study confirmed these published data on increased apoptosis and hence apoptotic bodies of cells incubated in calcifying conditions. CBF reversed or at least limited these processes significantly.

In addition to identifying a mediator of the complex pathobiology of vascular calcification, these findings have two potential clinical implications. First, we have identified a new mediator that regulates osteoblastic transdifferentiation and describes a new role for the adrenal medulla linking sympathetic nerve activity and arterial calcification. Second, the decreased plasma CBF concentration in patients suffering from increased vascular calcification may shed further light on the mechanisms of arterial calcification in renal failure. Modification of CBF release from adrenal chromaffin granules by sympathetic nerve stimulation might be an additional attractive approach to prevent or treat arterial calcification in these patients. However, the impact of the results of our current study has to be validated in larger clinical studies to clarify in detail the overall impact of the identified peptide regarding, e.g. protein loss via haemodialysis and/or the effect of CKD stage.

In conclusion, this study identifies a mediator of the differentiation of VSMCs and thus vascular calcification. This adrenal gland-derived compound may enhance our understanding of pathophysiological conditions related to calcification, such as calcific arteriopathy. Since CBF is secreted by adrenal chromaffin granules and presents at effective concentrations in human plasma, CBF is likely to participate in the regulation of vascular calcification and thus in reducing its harmful consequences.

With the identification and characterization of the CBF peptide, a hitherto unknown function of the adrenal glands in vascular calcification processes was detected. Due to the (patho-) physiological effect of CBF and its active amino acids, it also represents a promising basis for the development of a new drug for the prevention and therapy of vascular calcification in CKD patients. Beyond that, it also holds encouraging prospects for other patient groups in the future.

## Supplementary Information

Below is the link to the electronic supplementary material.

Supplementary file1 (PDF 914 KB)

## References

[1] Alexopoulos N, Raggi P (2009). Calcification in atherosclerosis. Nat Rev Cardiol.

[2] Balderman JA, Lee HY, Mahoney CE, Handy DE, White K, Annis S, Lebeche D, Hajjar RJ, Loscalzo J, Leopold JA (2012). Bone morphogenetic protein-2 decreases microRNA-30b and microRNA-30c to promote vascular smooth muscle cell calcification. J Am Heart Assoc.

[3] Baulmann J, Schillings U, Rickert S, Uen S, Dusing R, Illyes M, Cziraki A, Nickering G, Mengden T (2008). A new oscillometric method for assessment of arterial stiffness: comparison with tonometric and piezo-electronic methods. J Hypertens.

[4] Blacher J, Guerin AP, Pannier B, Marchais SJ, London GM (2001). Arterial calcifications, arterial stiffness, and cardiovascular risk in end-stage renal disease. Hypertension.

[5] Bokui N, Otani T, Igarashi K, Kaku J, Oda M, Nagaoka T, Seno M, Tatematsu K, Okajima T, Matsuzaki T, Ting K, Tanizawa K, Kuroda S (2008). Involvement of MAPK signaling molecules and Runx2 in the NELL1-induced osteoblastic differentiation. FEBS Lett.

[6] Bon N, Couasnay G, Bourgine A, Sourice S, Beck-Cormier S, Guicheux J, Beck L (2018). Phosphate (Pi)-regulated heterodimerization of the high-affinity sodium-dependent Pi transporters PiT1/Slc20a1 and PiT2/Slc20a2 underlies extracellular Pi sensing independently of Pi uptake. J Biol Chem.

[7] Bourgine A, Pilet P, Diouani S, Sourice S, Lesoeur J, Beck-Cormier S, Khoshniat S, Weiss P, Friedlander G, Guicheux J, Beck L (2013). Mice with hypomorphic expression of the sodium-phosphate cotransporter PiT1/Slc20a1 have an unexpected normal bone mineralization. PLoS ONE.

[8] Cerra MC, De Iuri L, Angelone T, Corti A, Tota B (2006). Recombinant N-terminal fragments of chromogranin-A modulate cardiac function of the Langendorff-perfused rat heart. Basic Res Cardiol.

[9] Chen G, Deng C, Li YP (2012). TGF-beta and BMP signaling in osteoblast differentiation and bone formation. Int J Biol Sci.

[10] Chen NX, Duan D, O'Neill KD, Moe SM (2006). High glucose increases the expression of Cbfa1 and BMP-2 and enhances the calcification of vascular smooth muscle cells. Nephrol Dial Transplant.

[11] Chen NX, Moe SM (2012). Vascular calcification: pathophysiology and risk factors. Curr Hypertens Rep.

[12] Chertow GM, Correa-Rotter R, Block GA, Drueke TB, Floege J, Goodman WG, Herzog CA, Kubo Y, London GM, Mahaffey KW, Mix TC, Moe SM, Wheeler DC, Parfrey PS (2012). Baseline characteristics of subjects enrolled in the Evaluation of Cinacalcet HCl Therapy to Lower Cardiovascular Events (EVOLVE) trial. Nephrol Dial Transplant.

[13] Cheung B, Leung R (1997). Elevated plasma levels of human adrenomedullin in cardiovascular, respiratory, hepatic and renal disorders. Clin Sci (Lond).

[14] Chirumamilla CS, Fazil M, Perez-Novo C, Rangarajan S, de Wijn R, Ramireddy P, Verma NK, Vanden Berghe W (2019). Profiling activity of cellular kinases in migrating T-cells. Methods Mol Biol.

[15] Collin-Osdoby P (2004). Regulation of vascular calcification by osteoclast regulatory factors RANKL and osteoprotegerin. Circ Res.

[16] Crouthamel MH, Lau WL, Leaf EM, Chavkin NW, Wallingford MC, Peterson DF, Li X, Liu Y, Chin MT, Levi M, Giachelli CM (2013). Sodium-dependent phosphate cotransporters and phosphate-induced calcification of vascular smooth muscle cells: redundant roles for PiT-1 and PiT-2. Arterioscler Thromb Vasc Biol.

[17] Derwall M, Malhotra R, Lai CS, Beppu Y, Aikawa E, Seehra JS, Zapol WM, Bloch KD, Yu PB (2012). Inhibition of bone morphogenetic protein signaling reduces vascular calcification and atherosclerosis. Arterioscler Thromb Vasc Biol.

[18] Durham AL, Speer MY, Scatena M, Giachelli CM, Shanahan CM (2018). Role of smooth muscle cells in vascular calcification: implications in atherosclerosis and arterial stiffness. Cardiovasc Res.

[19] Feng JQ, Xing L, Zhang JH, Zhao M, Horn D, Chan J, Boyce BF, Harris SE, Mundy GR, Chen D (2003). NF-kappaB specifically activates BMP-2 gene expression in growth plate chondrocytes in vivo and in a chondrocyte cell line in vitro. J Biol Chem.

[20] Ferrero E, Scabini S, Magni E, Foglieni C, Belloni D, Colombo B, Curnis F, Villa A, Ferrero ME, Corti A (2004). Chromogranin A protects vessels against tumor necrosis factor alpha-induced vascular leakage. FASEB J.

[21] Ferretti G, Bacchetti T, Negre-Salvayre A, Salvayre R, Dousset N, Curatola G (2006). Structural modifications of HDL and functional consequences. Atherosclerosis.

[22] Gaillard V, Casellas D, Seguin-Devaux C, Schohn H, Dauca M, Atkinson J, Lartaud I (2005). Pioglitazone improves aortic wall elasticity in a rat model of elastocalcinotic arteriosclerosis. Hypertension.

[23] Gaillard V, Jover B, Casellas D, Cordaillat M, Atkinson J, Lartaud I (2008). Renal function and structure in a rat model of arterial calcification and increased pulse pressure. Am J Physiol Renal Physiol.

[24] Gonzalez M, Martinez R, Amador C, Michea L (2009). Regulation of the sodium-phosphate cotransporter Pit-1 and its role in vascular calcification. Curr Vasc Pharmacol.

[25] Gupta N, Bark SJ, Lu WD, Taupenot L, O'Connor DT, Pevzner P, Hook V (2010). Mass spectrometry-based neuropeptidomics of secretory vesicles from human adrenal medullary pheochromocytoma reveals novel peptide products of prohormone processing. J Proteome Res.

[26] Hashimoto S, Ochs RL, Rosen F, Quach J, McCabe G, Solan J, Seegmiller JE, Terkeltaub R, Lotz M (1998). Chondrocyte-derived apoptotic bodies and calcification of articular cartilage. Proc Natl Acad Sci USA.

[27] Helle KB, Corti A, Metz-Boutigue MH, Tota B (2007). The endocrine role for chromogranin A: a prohormone for peptides with regulatory properties. Cell Mol Life Sci.

[28] Helle KB, Metz-Boutigue MH, Cerra MC, Angelone T (2018). Chromogranins: from discovery to current times. Pflugers Arch.

[29] Hilhorst R, Houkes L, Mommersteeg M, Musch J, van den Berg A, Ruijtenbeek R (2013). Peptide microarrays for profiling of serine/threonine kinase activity of recombinant kinases and lysates of cells and tissue samples. Methods Mol Biol.

[30] Hinson JP, Kapas S, Smith DM (2000). Adrenomedullin, a multifunctional regulatory peptide. Endocr Rev.

[31] Holmar J, Noels H, Bohm M, Bhargava S, Jankowski J, Orth-Alampour S (2020). Development, establishment and validation of in vitro and ex vivo assays of vascular calcification. Biochem Biophys Res Commun.

[32] Hruska KA, Mathew S, Saab G (2005). Bone morphogenetic proteins in vascular calcification. Circ Res.

[33] Huang YF, Lin JJ, Lin CH, Su Y, Hung SC (2012). c-Jun N-terminal kinase 1 negatively regulates osteoblastic differentiation induced by BMP2 via phosphorylation of Runx2 at Ser104. J Bone Miner Res.

[34] Jalili PR, Gheyi T, Dass C (2004). Proteome analysis in bovine adrenal medulla using matrix-assisted laser desorption/ionization mass spectrometry. Rapid Commun Mass Spectrom.

[35] Jankowski J, van der Giet M, Jankowski V, Schmidt S, Hemeier M, Mahn B, Giebing G, Tölle M, Luftmann H, Schlüter H, Zidek W, Tepel M (2003). Increased plasma phenylacetic acid in patients with end-stage renal failure inhibits iNOS expression. J Clin Invest.

[36] Jankowski V, Schulz A, Kretschmer A, Mischak H, van der Giet M, Janke D, Schuchardt M, Herwig R, Zidek W, Jankowski J (2013). The enzymatic activity of the VEGFR2-receptor for the biosynthesis of dinucleoside polyphosphates. J Mol Med.

[37] Jankowski V, Tölle M, Vanholder R, Schönfelder G, van der Giet M, Henning L, Schlüter H, Paul M, Zidek W, Jankowski J (2005). Identification of uridine adenosine tetraphosphate (Up_4_A) as an endothelium-derived vasoconstrictive factor. Nat Med.

[38] Jofre-Monseny L, Minihane AM, Rimbach G (2008). Impact of apoE genotype on oxidative stress, inflammation and disease risk. Mol Nutr Food Res.

[39] Kirton JP, Wilkinson FL, Canfield AE, Alexander MY (2006). Dexamethasone downregulates calcification-inhibitor molecules and accelerates osteogenic differentiation of vascular pericytes: implications for vascular calcification. Circ Res.

[40] Klein J, Eales J, Zurbig P, Vlahou A, Mischak H, Stevens R (2013). Proteasix: a tool for automated and large-scale prediction of proteases involved in naturally occurring peptide generation. Proteomics.

[41] Kork F, Jankowski J, Goswami A, Weis J, Brook G, Yamoah A, Anink J, Aronica E, Fritz S, Huck C, Schipke C, Peters O, Tepel M, Noels H, Jankowski V (2018). Golgin A4 in CSF and granulovacuolar degenerations of patients with Alzheimer disease. Neurology.

[42] Koumakis E, Millet-Botti J, Benna JE, Leroy C, Boitez V, Codogno P, Friedlander G, Forand A (2019). Novel function of PiT1/SLC20A1 in LPS-related inflammation and wound healing. Sci Rep.

[43] Kurnatowska I, Nowicki M (2006). Serum chromogranin A concentration and intradialytic hypotension in chronic haemodialysis patients. Int Urol Nephrol.

[44] Leopold JA (2015). Vascular calcification: mechanisms of vascular smooth muscle cell calcification. Trends Cardiovasc Med.

[45] Liu F, Zhong H, Liang JY, Fu P, Luo ZJ, Zhou L, Gou R, Huang J (2010). Effect of high glucose levels on the calcification of vascular smooth muscle cells by inducing osteoblastic differentiation and intracellular calcium deposition via BMP-2/Cbfalpha-1 pathway. J Zhejiang Univ Sci B.

[46] Liu X, Cao F, Liu S, Mi Y, Liu J (2018). BMP2/Smad signaling pathway is involved in the inhibition function of fibroblast growth factor 21 on vascular calcification. Biochem Biophys Res Commun.

[47] Liu Y, Shanahan CM (2011). Signalling pathways and vascular calcification. Front Biosci (Landmark Ed).

[48] Mikkelsen G, Asberg A, Hultstrom ME, Aasarod K, Hov GG (2017). Reference limits for chromogranin A, CYFRA 21–1, CA 125, CA 19–9 and carcinoembryonic antigen in patients with chronic kidney disease. Int J Biol Markers.

[49] Mohseni S, Emtenani S, Emtenani S, Asoodeh A (2014). Antioxidant properties of a human neuropeptide and its protective effect on free radical-induced DNA damage. J Pept Sci.

[50] Niederhoffer N, Bobryshev YV, Lartaud-Idjouadiene I, Giummelly P, Atkinson J (1997). Aortic calcification produced by vitamin D3 plus nicotine. J Vasc Res.

[51] Nurnberger J, Michalski R, Turk TR, Opazo Saez A, Witzke O, Kribben A (2011). Can arterial stiffness parameters be measured in the sitting position?. Hypertens Res.

[52] Oleson CV, Busconi BD, Baran DT (2002). Bone density in competitive figure skaters. Arch Phys Med Rehabil.

[53] Oppi S, Luscher TF, Stein S (2019). Mouse models for atherosclerosis research-which is my line?. Front Cardiovasc Med.

[54] Ozkok A, Caliskan Y, Sakaci T, Erten G, Karahan G, Ozel A, Unsal A, Yildiz A (2012). Osteoprotegerin/RANKL axis and progression of coronary artery calcification in hemodialysis patients. Clin J Am Soc Nephrol.

[55] Proudfoot D, Skepper JN, Hegyi L, Bennett MR, Shanahan CM, Weissberg PL (2000). Apoptosis regulates human vascular calcification in vitro: evidence for initiation of vascular calcification by apoptotic bodies. Circ Res.

[56] Rao N, Crail S (2013). Images in clinical medicine. Metastatic calcification and long-term hemodialysis. N Engl J Med.

[57] Roatta S, Passatore M, Novello M, Colombo B, Dondossola E, Mohammed M, Losano G, Corti A, Helle KB (2011). The chromogranin A- derived N-terminal peptide vasostatin-I: In vivo effects on cardiovascular variables in the rabbit. Regul Pept.

[58] Rong S, Zhao X, Jin X, Zhang Z, Chen L, Zhu Y, Yuan W (2014). Vascular calcification in chronic kidney disease is induced by bone morphogenetic protein-2 via a mechanism involving the Wnt/beta-catenin pathway. Cell Physiol Biochem.

[59] Sage AP, Tintut Y, Demer LL (2010). Regulatory mechanisms in vascular calcification. Nat Rev Cardiol.

[60] Sahu BS, Sonawane PJ, Mahapatra NR (2010). Chromogranin A: a novel susceptibility gene for essential hypertension. Cell Mol Life Sci.

[61] Salem S, Jankowski V, Asare Y, Liehn E, Welker P, Raya-Bermudez A, Pineda-Martos C, Rodriguez M, Munoz-Castaneda JR, Bruck H, Marx N, Machado FB, Staudt M, Heinze G, Zidek W, Jankowski J (2015). Identification of the “vasoconstriction inhibiting factor” (VIF), a potent endogenous cofactor of angiotensin II acting on the AT2 receptor. Circulation.

[62] Schillaci G, De Vuono S, Pucci G (2011). An endogenous brake on the sympathetic nervous system: the emerging role of catestatin in hypertension. J Cardiovasc Med (Hagerstown).

[63] Schlieper G, Schurgers L, Brandenburg V, Reutelingsperger C, Floege J (2016). Vascular calcification in chronic kidney disease: an update. Nephrol Dial Transplant.

[64] Schulz AM, Terne C, Jankowski V, Cohen G, Schaefer M, Boehringer F, Tepel M, Kunkel D, Zidek W, Jankowski J (2014). Modulation of NADPH oxidase activity by known uraemic retention solutes. Eur J Clin Invest.

[65] Servin-Gonzalez LS, Granados-Lopez AJ, Lopez JA (2015). Families of microRNAs expressed in clusters regulate cell signaling in cervical cancer. Int J Mol Sci.

[66] Son BK, Kozaki K, Iijima K, Eto M, Nakano T, Akishita M, Ouchi Y (2007). Gas6/Axl-PI3K/Akt pathway plays a central role in the effect of statins on inorganic phosphate-induced calcification of vascular smooth muscle cells. Eur J Pharmacol.

[67] Tagliabracci VS, Engel JL, Wen J, Wiley SE, Worby CA, Kinch LN, Xiao J, Grishin NV, Dixon JE (2012). Secreted kinase phosphorylates extracellular proteins that regulate biomineralization. Science.

[68] Tesauro M, Mauriello A, Rovella V, Annicchiarico-Petruzzelli M, Cardillo C, Melino G, Di Daniele N (2017). Arterial ageing: from endothelial dysfunction to vascular calcification. J Intern Med.

[69] Tota B, Quintieri AM, Di Felice V, Cerra MC (2007). New biological aspects of chromogranin A-derived peptides: focus on vasostatins. Comp Biochem Physiol A Mol Integr Physiol.

[70] Tousoulis D, Siasos G, Maniatis K, Oikonomou E, Vlasis K, Papavassiliou AG, Stefanadis C (2012). Novel biomarkers assessing the calcium deposition in coronary artery disease. Curr Med Chem.

[71] Towler DA, Shao JS, Cheng SL, Pingsterhaus JM, Loewy AP (2006). Osteogenic regulation of vascular calcification. Ann N Y Acad Sci.

[72] Vaingankar SM, Li Y, Biswas N, Gayen J, Choksi S, Rao F, Ziegler MG, Mahata SK, O'Connor DT (2010). Effects of chromogranin A deficiency and excess in vivo: biphasic blood pressure and catecholamine responses. J Hypertens.

[73] van der Giet M, Khattab M, Börgel J, Schlüter H, Zidek W (1997). Differential effects of diadenosine phosphates on purinoceptors in the rat isolated perfused kidney. Br J Pharmacol.

[74] Vanholder R, De Smet R, Glorieux G, Argiles A, Baurmeister U, Brunet P, Clark W, Cohen G, De Deyn PP, Deppisch R, Descamps-Latscha B, Henle T, Jorres A, Lemke HD, Massy ZA, Passlick-Deetjen J, Rodriguez M, Stegmayr B, Stenvinkel P, Tetta C, Wanner C, Zidek W (2003). Review on uremic toxins: classification, concentration, and interindividual variability. Kidney Int.

[75] Wegrzyn J, Lee J, Neveu JM, Lane WS, Hook V (2007). Proteomics of neuroendocrine secretory vesicles reveal distinct functional systems for biosynthesis and exocytosis of peptide hormones and neurotransmitters. J Proteome Res.

[76] Wei Z, Biswas N, Wang L, Courel M, Zhang K, Soler-Jover A, Taupenot L, O'Connor DT (2011). A common genetic variant in the 3'-UTR of vacuolar H+-ATPase ATP6V0A1 creates a micro-RNA motif to alter chromogranin A processing and hypertension risk. Circ Cardiovasc Genet.

[77] Willems BA, Furmanik M, Caron MMJ, Chatrou MLL, Kusters DHM, Welting TJM, Stock M, Rafael MS, Viegas CSB, Simes DC, Vermeer C, Reutelingsperger CPM, Schurgers LJ (2018). Ucma/GRP inhibits phosphate-induced vascular smooth muscle cell calcification via SMAD-dependent BMP signalling. Sci Rep.

[78] Xie H, Xie PL, Wu XP, Chen SM, Zhou HD, Yuan LQ, Sheng ZF, Tang SY, Luo XH, Liao EY (2011). Omentin-1 attenuates arterial calcification and bone loss in osteoprotegerin-deficient mice by inhibition of RANKL expression. Cardiovasc Res.

[79] Yang B, Lin X, Yang C, Tan J, Li W, Kuang H (2015). Sambucus Williamsii Hance promotes MC3T3-E1 cells proliferation and differentiation via BMP-2/Smad/p38/JNK/Runx2 signaling pathway. Phytother Res.

[80] Yao Y, Watson AD, Ji S, Bostrom KI (2009). Heat shock protein 70 enhances vascular bone morphogenetic protein-4 signaling by binding matrix Gla protein. Circ Res.

[81] Yener S, Ertilav S, Secil M, Akinci B, Demir T, Comlekci A, Yesil S (2009). Natural course of benign adrenal incidentalomas in subjects with extra-adrenal malignancy. Endocrine.

[82] Zhang D, Bi X, Liu Y, Huang Y, Xiong J, Xu X, Xiao T, Yu Y, Jiang W, Huang Y, Zhang J, Zhang B, Zhao J (2017). High phosphate-induced calcification of vascular smooth muscle cells is associated with the TLR4/NF-kappab signaling pathway. Kidney Blood Press Res.

[83] Zhao G, Xu MJ, Zhao MM, Dai XY, Kong W, Wilson GM, Guan Y, Wang CY, Wang X (2012). Activation of nuclear factor-kappa B accelerates vascular calcification by inhibiting ankylosis protein homolog expression. Kidney Int.

[84] Zhu D, Deng X, Han XF, Sun XX, Pan TW, Zheng LP, Liu YQ (2018). Wedelolactone enhances osteoblastogenesis through ERK- and JNK-mediated BMP2 expression and Smad/1/5/8 phosphorylation. Molecules.

