# Supplementary material for: Prevention of vascular calcification by the endogenous chromogranin A-derived mediator that inhibits osteogenic transdifferentiation
Source: Basic Res Cardiol. 2021 Oct 13;116(1):57. doi: 10.1007/s00395-021-00899-z (PMC8514386; doi:10.1007/s00395-021-00899-z)
Supplement: Supplementary file 1 — Supplementary file1 (PDF 914 KB) [file 395_2021_899_MOESM1_ESM.pdf]

Basic Research in Cardiology
May 05th, 2021
Revised version, July 17th, 2021
2nd revised version, September 14th,2021
EMID: b9a699ddb1a24692

Prevention of vascular calcification by the endogenous chromogranin A-derived mediator that inhibits osteogenic transdifferentiation

Setareh Orth-Alampour[1)*)], Nathalie Gayrard[2)*)], Silvia Salem[3)*)], Shruti Bhargava[1)], Vera Jankowski[1)], Bernard Jover[4)], Cécile Notarnicola[4)], Heidi Noels[1)], Emiel P.C. van der Vorst[1)], Christoph Kuppe[11)], Michael Wolf [12)], Claudia Goettsch[8)], Wendy Theelen[1)], Heike Bruck[5)], Danilo Fliser[6)], Joseph Loscalzo[7)], Zhuojun Wu[1)], Nikolaus Marx[8)], Walter Zidek[9)], Àngel Argilés[2)**)], and Joachim Jankowski[1)10)**)]

*) and **): These authors contributed equally

[1)] Institute of Molecular Cardiovascular Research, Medical Faculty, RWTH Aachen University, Germany

[2)] RD-Néphrologie & EA7288 University of Montpellier, France

[3)] Clinic for Pediatric Cardiology & Intensive Care, Medical School, Hannover, Germany

[4)] Physiology & Experimental Medicine Heart Muscles, INSERM-CNRS-Université Montpellier, France

[5)] University Hospital Essen, Department of Nephrology, Essen, Germany & Helios Clinic Krefeld; Medical Clinic III, Krefeld, Germany

[6)] Universitätsklinikum Homburg, Medizinische Klinik IV, Homburg, Germany

[7)] Department of Medicine, Brigham and Women’s Hospital, Harvard Medical School, Boston, Massachusetts, USA

[8)] Medical Clinic I, Medical Faculty, RWTH Aachen University, Germany

[9)] Meoclinic, Department of Nephrology & Charité, Department of Nephrology, Berlin, Germany

[10)] Experimental Vascular Pathology, Cardiovascular Research Institute Maastricht (CARIM), University of Maastricht, The Netherlands

[11)] Institute of Experimental Medicine and Systems Biology, RWTH Aachen University, Germany

[12)] Department of Orthodontics, Medical Faculty, RWTH Aachen University, Germany

ADDRESS FOR CORRESPONDENCE

Univ.-Prof. Dr. J. Jankowski
RWTH University of Aachen | Institute of Molecular Cardiovascular Research
Pauwelsstrasse 30 | 52074, Aachen | Germany
Tel.: (+49)-241-80-8050 | Fax: (+49)-241-80-52716
E-mail: jjankowski@ukaachen.de

Orth-Alampour *et al.* Supplementary figure 1

a

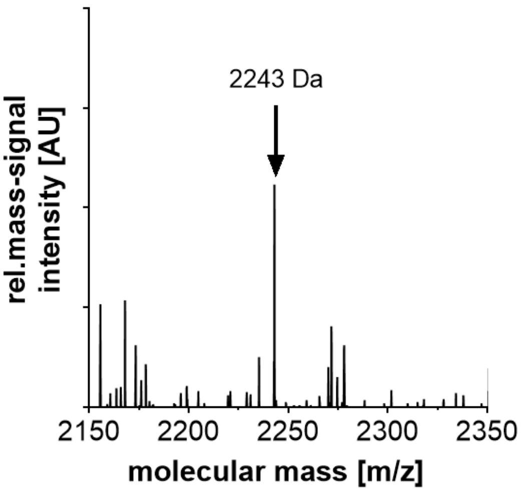

b

MRSAAVLALLLCAGQVTALPVNSPMNKGDTEVMKCIVEVISDTL
SKPSPMPVSQECFETLRGDERILSILRHQNLLKELQDLALQGAK
ERAHQQKKHSGFEDELSEVLENQSSQAELKEAVEEPSSKDV
MEKREDSKEAEKSGEATDGARPQALPEPMQESKAEGNNQAP
GEEEEEEEEATNTHPPASLPSQKYPGPQAEGDSEGLSQGLVD
REKGLSAEPGWQAKREEEEEEEEEEAEAGEEAVPEEEGPTVV
LNPHPSLGYKEIRKGESRSEALAVDGAGKPGAEEAQDPEGKG
EQEHSQQKEEEEEMAVVPQGLFRGGKSGELEQEEERSKEW
EDSKRWSKMDQLAKELTAEKR**LEGQEEEEDNRDSSMKLSF**RA
RAYGFRGPGPQLRRGWRPSSREDSLEAGLPLQVRGYPEEKK
EEEGSANRRPEDQELESLSAIEAELEKVAHQLQALRRG

c

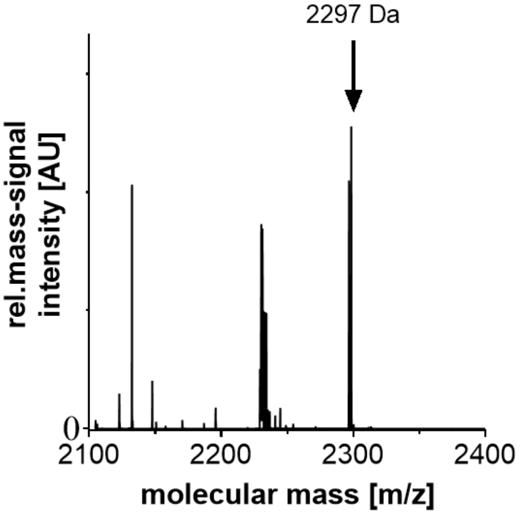

Orth-Alampour *et al.* Supplementary Figure 2

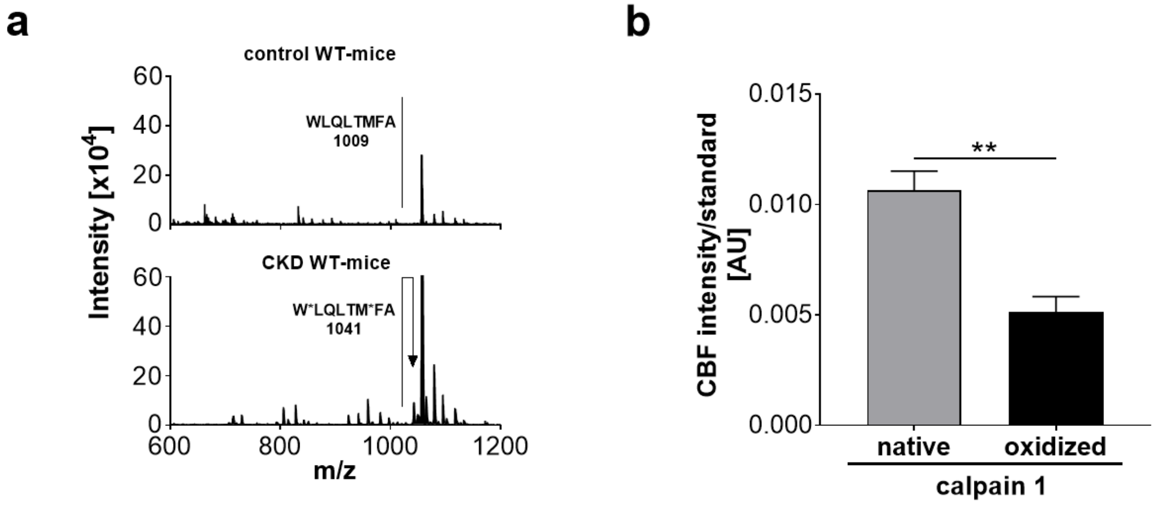

Orth-Alampour *et al.* Supplementary figure 3

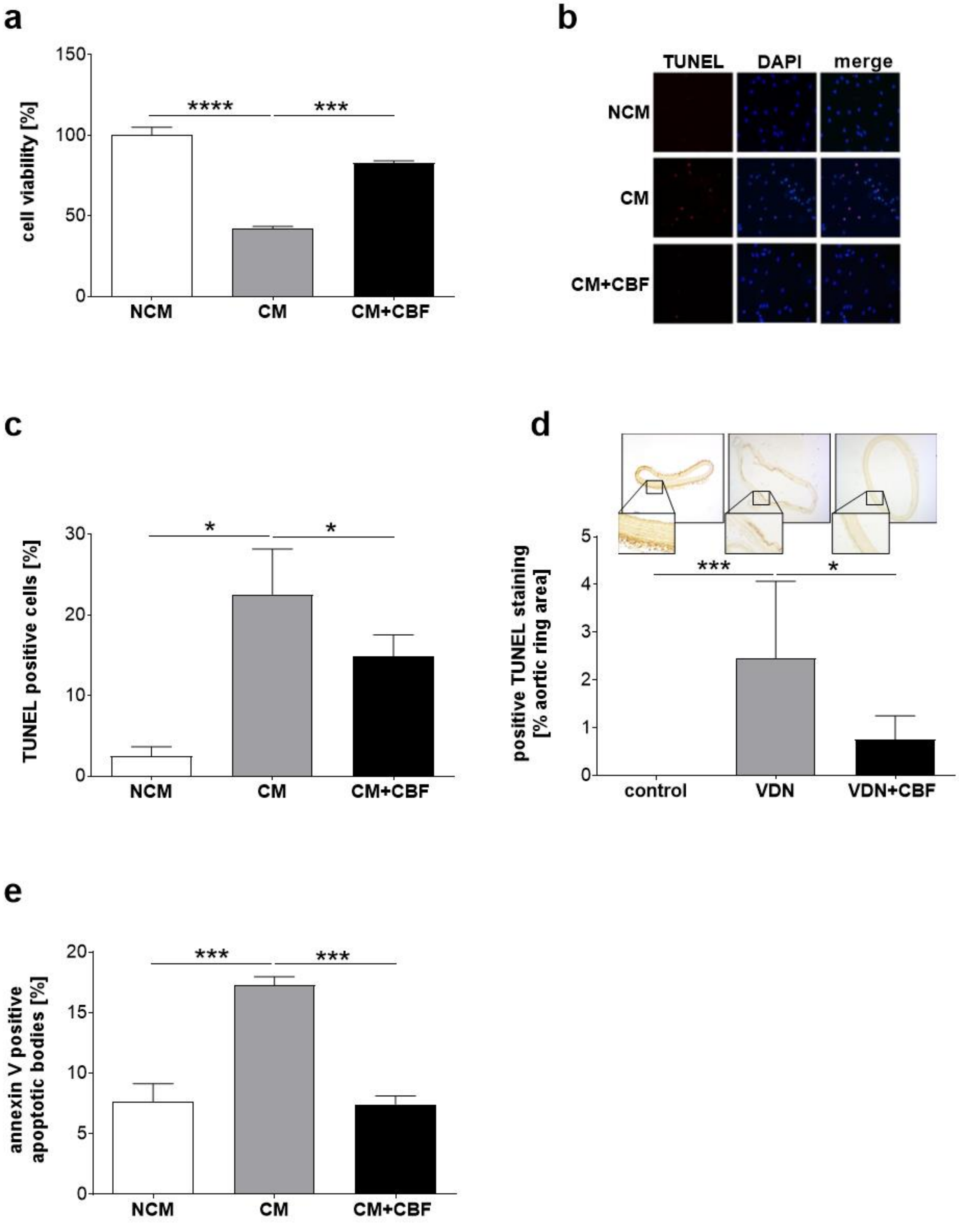

Orth-Alampour *et al.* Supplementary figure 4

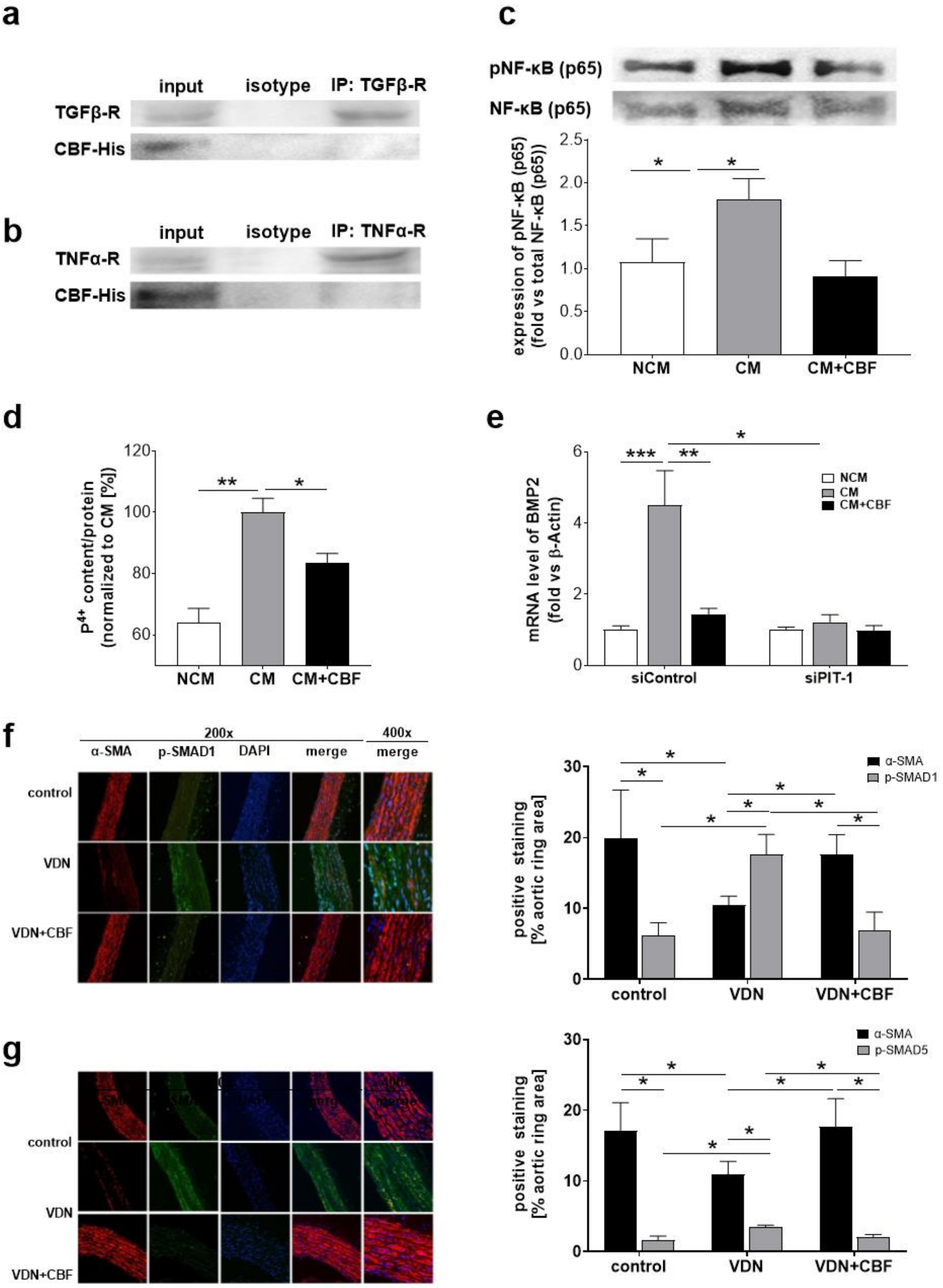

Supplementary Table 1: Clinical and biochemical characteristics of ESRD patients whose CBF concentrations were determined before and after hemofiltration treatment (values are mean ± SEM).

**Parameter**	**ESRD patients (N = 10)**
age (years)	56.0 ± 3.9
blood pressure (mmHg)	
systolic	133.1 ± 3.6
diastolic	68.5 ± 3.6
weight (kg)	75.5 ± 8.2
BMI (kg m^{-2})	25.8 ± 2.9
PTH (pg ml^{-1})	323.4 ± 86.5
serum calcium (mg dl^{-1})	4.4 ± 0.1
serum phosphate (mg dl^{-1})	5.6 ± 0.4
C-reactive protein (mg/dl)	0.8 ± 0.2
magnesium (mg/dl)	2.7 ± 0.3
time on HDF (months)	50.3 ± 17.1

SUPPLEMENTARY FIGURES LEGENDS

Supplementary Figure 1: Identification of human CBF from plasma

(a) Electrospray ionization-mass spectrum of a chromatographic fraction isolated from human plasma. The mass-signal (m/z) at 2,243 Da $[M+H]^+$ corresponds to the human amino acid sequence of the CBF peptide (LEGQEEEEDNRDSSMKLSF).

(b) The peptide sequence of CBF represented in black characters matches the 358-376 amino acid position of (human chromogranin A protein represented in grey characters).

(c) MALDI-TOF mass spectrum of a fraction isolated from adrenal gland chromaffin granules. The mass signal at m/z 2,297 Da $(M+H^+)$ belongs to *CBF*.

Supplementary Figure 2: The effect of post-translational modification of calpain 1 on CBF release from elongated CBF

(a) Characteristic MALDI mass spectra of the chromogranin A cleaving enzyme calpain 1 isolated from control C57BL/6J wildtype mice (upper panel) and CKD adenine C57BL/6J wildtype mice (lower panel) demonstrating CKD-associated enzyme modification by oxidation. The induction of CKD was performed by a 1.5 weeks diet of 0.3% adenine. A stable CKD condition was subsequently achieved with 0.2% adenine for 6.5 weeks. (N=5 per group).

(b) LC-qTOF mass-spectrometric analyses of the elongated CBF incubated in the presence of native calpain 1 (grey bar) or the presence of oxidized calpain 1 (black bar). The relative mass signal of CBF intensity was normalized to the standard. Data are shown as mean ± SEM. **P≤0.001 compared with native calpain 1 based on unpaired t-tests (N=3 in each group).

Supplementary Figure 3: CBF inhibits apoptosis in Smooth muscle cells

(a) Quantification of cell viability of cultivated human aortic smooth muscle cells incubated in non-calcifying conditions (non-calcifying medium (NCM)) and under calcifying conditions in the absence (calcifying medium (CM)) or presence of CBF (CM+CBF) for 4 days using MTT assay using 3-(4,5-dimethyl-2-thiazolyl)-2, 5-diphenyl-2H-tetrazolium bromide as a dye. Data are shown as mean ± SEM. ***P≤0.0001, ****P≤0.00=01 compared with CM group based on one-way ANOVA. Bonferroni's multiple comparisons were used as a post-test (N=3 per group).

(b-c) Representative picture (b) and quantification (c) of cell apoptosis in cultivated human aortic smooth muscle cells incubated in non-calcifying conditions (non-calcifying medium

(NCM)) and under calcifying conditions in the absence (calcifying medium (CM)) or presence of CBF (CM+CBF) for 4 days using "terminal deoxynucleotidyl transferase dUTP nick end labelling" (TUNEL) staining. Data are shown as mean ± SEM. *P<0.05 compared with CM group based on one-way ANOVA. Bonferroni's multiple comparisons were used as a post-test (N=3 per group).

(d) Quantification of cell apoptosis in thoracic aortic rings isolated from Wistar control rats (control), and VDN rats untreated (VDN) or treated with CBF (VDN+CBF)) by TUNEL staining. Data are shown as mean ± SEM. *P<0.05, ***P≤0.0001 compared with VDN group based on one-way ANOVA. Bonferroni's multiple comparisons were used as a post-test (N=6 per group).

(e) Quantification of annexin V positive apoptotic bodies in the supernatant of cultivated human aortic smooth muscle cells incubated in non-calcifying conditions (non-calcifying medium (NCM)) and under calcifying conditions in the absence (calcifying medium (CM)) or presence of CBF (CM+CBF) for 5 days using FACS analysis. Data are shown as mean ± SEM. ***P≤0.0001 compared with the VDN group based on one-way ANOVA. Bonferroni's multiple comparisons were used as a post-test (N=4 per group).

Supplementary Figure 4: CBF inhibits phospho-SMAD1/5 in *ex vivo*

(a-b) Co-immunoprecipitation of TGFβ-receptor and CBF-His tag (a) and TNFα-receptor and CBF-His tag (b) using HAoSMCs cell lysate. A representative Western blot image is shown (N=3).

(c) Western blot and quantitative analysis of NF-κB (p65) activation of cultivated human aortic smooth muscle cells incubated under non-calcifying conditions (non-calcifying medium (NCM; white bar)) and calcifying conditions in the absence (CM; grey bar) or presence of CBF (black bar). Data are shown as mean ± SEM. *P<0.05 compared with CM group

based on one-way ANOVA. Bonferroni's multiple comparisons were used as a post-test (N=6 per group).

(d) Quantification of phosphate (P^{4+}) content of VSMCs incubated under non-calcifying conditions (NCM; white bar) and calcifying conditions in the absence (CM; grey bar) or presence of CBF (100 nmol L^{-1}) (CM+CBF; black bar), respectively. *P<0.05 and **P≤0.001 compared with CM based on one-way ANOVA. Bonferroni's multiple comparisons were used as a post-test (N=4 per group).

(e) Quantification of bone morphogenetic protein 2 (BMP2) gene expression in VSMCs transfected with a control and PIT-1 siRNA, respectively, after incubation under non-calcifying conditions (NCM; white bar) or calcifying conditions in the absence (CM; grey bar) or presence of CBF (black bar). Data are shown as mean ± SEM. *P<0.05, **P≤0.001, ***P≤0.0001. The data based on one-way ANOVA. Bonferroni's multiple comparisons were used as a post-test (N=5 per group).

(f-g) Relative quantification of (**a**) α-SMA and phospho-SMAD1 (P-smad1) and (**b**) α-SMA and phospho-SMAD5 (p-SMAD5) of thoracic aortic rings of control rats (white bar), untreated VDN rats (grey bar) or treated VDN rats with CBF (31µg kg-1 per day for 4 weeks) (black bar). Representative images (original magnification x 200, Scale bar 200 µm) of (**a**) α-SMA (red) and p-SMAD1 and (**b**) α-SMA (red) and p-SMAD5 (green), DAPI staining of nuclei (blue), and corresponding merged images are shown. Data are shown as mean ± SEM. *P<0.05. The data based on one-way ANOVA. Bonferroni's multiple comparisons were used as a post-test (N=7 in each group).

SUPPLEMENTARY METHODS / ONLINE METHODS

CHEMICALS

HPLC water (gradient grade), acetonitrile (ACN), magnesium chloride-hexahydrate and disodium hydrogen phosphate (Na_2HPO_4) were purchased from Merck. Sodium dihydrogen phosphate

(NaH_2PO_4) and sodium hydroxide (NaOH) were purchased from Roth. All other substances were obtained from Sigma-Aldrich.

MECHANICAL DISINTEGRATION AND EXTRACTION

Fresh bovine adrenal glands were obtained on ice from a local slaughterhouse, and the lipid matter was removed. Adrenal glands were then cut into pieces of about 1 g snap-frozen in liquid nitrogen for 30 min and stored at -80°C for 12 h followed by lyophilisation. The freeze-dried adrenal glands were then mechanically pulverized at 4°C. Seventy ml of 0.6 mmol L^{-1} perchloric acid (T=4°C) was added to 700 g of pulverized adrenal glands. The resulting mixture was homogenised with an Ultra-Turrax (9.500 g min^{-1}) for 1 min at 4°C. This procedure was repeated 10 times. The homogenate was centrifuged at 2000 g for 15 min at 4°C. The supernatant's pH was adjusted to 9.0 - 11.0 by adding an aqueous saturated KOH solution. The supernatant was stored at -20°C for at least 12 h to precipitate $KClO_4$.

PREPARATIVE REVERSED-PHASE CHROMATOGRAPHY

The supernatant of the homogenate was adjusted to pH 6.5 by 0.1 mol L^{-1} HCl, and the samples were loaded onto a reversed-phase chromatography column (LiChroprep RP C 18, 310 x 25 mm, Merck). The chromatographic step was performed on a BioRad BioLogic DuoFlow HPLC device with UV-detection at 280 nm; 0.1% trifluoroacetic acid (TFA) in water was used as eluent A and 80% acetonitrile in water was used as eluent B with a flow rate of 3 ml min^{-1}. A stepwise gradient (20, 40, 60, 80, and 100% eluent B) was used to elute the retained substances from the chromatographic column. The fractions were lyophilized and then resuspended in 20 mmol L^{-1} K_2HPO_4 in water.

EFFECT OF CHROMATOGRAPHIC FRACTIONS ON *EX VIVO* THORACIC AORTIC VASCULAR CALCIFICATION

For analysing the effect of chromatographic fractions on vascular calcification processes recently published methods were used [4]. Briefly, aortas from adult male Wistar rats were gently dissected and divided into segments (length: 3-4 mm). The thoracic aortic rings were incubated with calcifying medium (Dulbecco's modified Eagle's medium containing 2.8 mmol L^{-1} phosphate) in the presence of chromatographic fractions of the adrenal glands. The thoracic aortic rings were cultured for 7 days at 37°C in a humidified atmosphere containing 5% CO_2 and media was changed every two days. Calcium content was determined as described below.

ANION-EXCHANGE CHROMATOGRAPHY

The lyophilized fractions were resuspended in 20 mmol L^{-1} K_2HPO_4 in water, and the pH was adjusted to 8 with 0.1 mol L^{-1} NaOH or 0.1 mol L^{-1} HCl. The eluate was further fractionated by preparative anion exchange chromatography (column: Superformance™ 16, 150 ml, 30 cm x 1.6 cm, gel: Fractogel EMP TMAE™; both Merck, Darmstadt, Germany). Twenty mmol L^{-1} K_2HPO_4 in water was used as eluent A; 1 mol L^{-1} NaCl in K_2HPO_4 (20 mmol L^{-1} in water) was used as eluent B with a flow rate of 1 ml min^{-1}. The retained substances were eluted from the chromatographic column using a stepwise gradient (20, 40, 60, 80, and 100% eluent B), with detection at 280 nm.

REVERSED-PHASE CHROMATOGRAPHY

The eluate from the anion exchange column was adjusted to pH 6 with 0.1 mol L^{-1} NaOH or by 0.1 mol L^{-1} HCl. One hundred µl of 1 mol L^{-1} trifluoroacetic acid (TFA) was added to each fraction of the anion exchange eluate. Thereafter, the samples were loaded onto a reversed-phase chromatographic column (LiChroprep RP C 18, 310 x 25 mm, Merck); 0.1% TFA in water (1:1000, v/v; eluent A) was used for equilibration (flow rate: 3 ml min^{-1}), and 80% acetonitrile in water (80:20, v/v; eluent B) and a stepwise gradient using 20, 40, 60, 80, and 100% eluent B was used for elution (flow rate: 3 ml min^{-1}). The eluate was lyophilized and stored at 20°C until further fractionation. Thereafter, the lyophilized fractions were resuspended in 15 ml H_2O and were fractionated by a reversed-phase chromatographic column (LiChroprep RP C 18e, 100 x 4.6 mm, Merck).

Once again, 0.1% trifluoroacetic acid (TFA) in water (1:1000, v/v) was used as eluent A and 80% acetonitrile in water (80:20, v/v) as eluent B; a linear gradient (1% to 100% ACN in 90 min; flow rate: 1 ml min^{-1}) was used for fractionation.

The chromatographic purification was performed on a BioLogic DuoFlow HPLC device (Biorad) with a UV detector at 280 nm. The eluate fractions were lyophilized and were resuspended in 10 µl H_2O. Each fraction of the chromatographic procedure was again screened for inhibitory effects on calcification by using aortic culture and a calcification assay.

MATRIX-ASSISTED LASER DESORPTION/IONIZATION MASS SPECTROMETRY

The lyophilized fractions from the reversed-phase chromatography were analysed by matrix-assisted laser desorption/ionization mass spectrometry (MALDI-MS) and MALDI-TOF/TOF fragment ion analysis. The lyophilized fractions were resuspended in 10 µl H_2O. One µl of each fraction was prepared on a prestructured MALDI sample support (MTP AnchorChip™ 400/384, Bruker Daltonics, Bremen, Germany) using 2,5-dihydroxybenzoic acid (DHB) affinity sample preparation [6]. Mass-spectrometric measurements were performed on a Bruker Ultraflex-III TOF/TOF instrument (Bruker-Daltonics). The instrument was equipped with a Smart beam™ laser operating with a repetition rate of 100-200 Hz. On average, the presented spectra represent the sum of 200 single-shot spectra for MS mode and 600 for MS/MS mode. Mass-spectra of positively charged ions were analysed in the reflector mode using delayed ion extraction. Fragment ion spectra were recorded using the LIFT option of the instrument. The calibration constants were determined using standard peptides prepared on positions adjacent to the sample, resulting in an error of <100 ppm for the recorded mass spectra. Peptide identification using the obtained fragment ion mass data was performed using the Mascot search engine (Matrix Science) as well as the RapideNovo 3.0.1 sequencing Tool (Bruker-Daltonic).

ELECTROSPRAY-IONIZATION-MASS-SPECTROMETRY (ESI-MS)

The MS/MS fragmentation analysis was also performed by liquid-chromatography/ electrospray-mass-spectrometry (ESI). Two capillary HPLC-pumps (G1376A, Agilent) with micro vacuum degasser (G1379B, Agilent), an automatic micro-well-plate autosampler (G1377A, Agilent), and a diode array multi-wavelength detector (G1365D Agilent) were used for chromatography. Chromatography was carried out using an Agilent Zorbax SB-C18 column (5 µm, 150 x 0.5 mm, Agilent), the column temperature was adjusted at 25°C, and 0.01% TFA and 0.1% formic acid (FA) in water was used as eluent A with 98% acetonitrile in water used as eluent B in an injection volume of 5 µl. A flow rate of 10 µl min^{-1} and a linear gradient (0 min: 1% eluent B; 30 min: 60% eluent B) were used for elution of retained substances from the column. The ESI-mass-spectrometry was performed in enhanced positive mode. The range of m/z was 80 to 2800. The scan speed of measured mass-spectra was 26,000 m/z per second. The accumulation time was adjusted to 200 ms. The flow rate of the nitrogen gas was 5 L min^{-1}. The temperature of the transfer capillary was adjusted to 300°C with the capillary voltage at 1,500 to 4,500 V. The mass-spectrometric data were accumulated by the HyStar software 3.2 (Bruker-Daltonics) and analysed by Data Analysis 4.0 (Bruker-Daltonics). Glutathione was used as an internal standard for the calculation of the recovery rate.

PEPTIDE SYNTHESIS

"Calcification blocking factor" (CBF) was synthesized automatically by the solid-phase method using standard Fmoc chemistry in continuous flow mode TentaGel S Random-Access Memory (RAM) resin 0.21 mmol g^{-1} for peptide amides, TentaGel S p-hydroxybenzoic acid (PHB) resin (Rapp Polymere) for the free acid of urocortin, o-benzotriazole-N, N, N', N'-tetramethyl- uronium-hexafluoro-phosphate (HBTU), 2 equivalents of N, N-diisopropylethylamine (DIEA), coupling for 20 min, deblocking with 20% piperidine in N, N-dimethyl formamide (DMF) for 15 min, and final cleavage with 95% TFA/5% water for 3 h. Purification of the crude peptide was carried out by preparative HPLC on PolyEncap A300 (10 µm particle size, 250 mm x 20 mm, Bischoff Analysentechnik) in water with increasing concentrations of ACN as mobile phase. An eluent gradient of

5-70 (v/v-%) ACN/water (0.1% TFA) over 70 min with a flow rate of 10 ml min^{-1} was used. The purified peptide was lyophilized. The peptide was characterized by MALDI mass-spectroscopy on a Voyager-DE STR BioSpectrometry Workstation MALDI-TOF mass-spectrometer (Perseptive Biosystems) using R-cyano-4- hydroxycinnamic acid and sinapinic acid as matrix and gave the expected $(M + H)^+$ mass [2].

ISOLATION AND STIMULATION OF ADRENAL GLAND GRANULES BY CARBACHOL

Fresh bovine adrenal glands were obtained on ice from a local slaughterhouse, the lipid matter was removed, and the adrenal glands were dissected. The granules were isolated from the adrenal glands as follows [1, 10]. Briefly, 50 ml of 0.32 mol L^{-1} sucrose were added to the adrenal glands, which were then homogenized with an Ultra-Turrax at 4°C (750 rpm) for 4 min, and the homogenate centrifuged at 800 g at 4°C for 10 min. The supernatant was centrifuged again at 10,000 g for 20 min at 4°C. To the supernatant, 50 ml of 1.6 mol L^{-1} sucrose was added, and then the sample was centrifuged at 100,000 g for 45 min at 4°C. The pellet was resuspended in 10 ml of water and divided into two aliquots. For an active release of CBF from the granules, carbachol (10^{-5} mol L^{-1}) was added to one aliquot. Phosphate-buffered saline (PBS, Sigma-Aldrich) was added to the second aliquot as a negative control and vortexed for 5 min at room temperature. The supernatant was analysed by electrospray-ionization mass-spectrometry as described above.

IDENTIFICATION OF ENZYMES CLEAVING CBF FROM CHROMOGRANIN A

The protease prediction tool Proteasix [7] was used to predict the enzymes likely involved in cleaving CBF from chromogranin A. Enzyme candidates were identified by entering the Protein ID P10645, the N-terminal amino acid 358 and C-terminal amino acid 376. Peptides with ten additional amino acids at the N-terminus of CBF (LAKELTAEKR-LEGQEEEEDNRDSSMKLSF) and

ten additional amino acids at the C-terminus (LEGQEEEEDNRDSSMKLSF-RARAYGFRGP), respectively, were synthesized for identification of the enzymes cleaving the N-terminal and C-terminal part of CBF, respectively, from chromogranin A.

Human kallikrein (9 µmol L^{-1}; Sigma-Aldrich) and the peptide with the amino acid sequence LAKELTAEKR-LEGQEEEEDNRDSSMKLSF (10 µmol L^{-1}; Campro, Berlin, Germany) were incubated in 1 ml PBS for 48 h at 37°C. As a control, LAKELTAEKR-LEGQEEEEDNRDSSMKLSF (10 µmol L^{-1}) was simultaneously incubated in the absence of enzymes. The reaction mixtures were deproteinized using 70% perchloric acid (Merck) at a final concentration of 0.6 mol L^{-1}, centrifuged at 13,000 g for 10 min at 4°C, the pH value was adjusted to 9 by using KOH, and the sample was analysed by LC-qTOF mass-spectrometry as described above. Calpain 1 (Sigma-Aldrich) was dialyzed using a Pur-A-Lyzer Maxi 12000 dialysis kit (Sigma-Aldrich) for removing EDTA and EGTA from the mixture. Afterwards, calpain 1 (6 µmol L^{-1}) and the peptide with the amino acid sequence LEGQEEEEDNRDSSMKLSF-RARAYGFRGP (1.5 µmol L^{-1}; Campro) were incubated in 1 ml PBS for 48 h at 37°C. The protein was removed from the reaction mixture using an Amicon Ultra 10K Centrifugal Filter (Merck, Darmstadt, Germany) and the filtrate was analysed by LC-qTOF mass-spectrometry as described above. Finally, calpain 1 and kallikrein at the same concentrations as mentioned previously and chromogranin A (1 µmol L^{-1}; Sigma-Aldrich) were incubated in 1 ml PBS for 48 h at 37°C. The enzymes were removed from the reaction mixture using an Amicon Ultra 10K Centrifugal Filter (Merck) and the filtrate was analysed by LC-qTOF mass-spectrometry as described above.

The reaction mixtures were fractionated by using a nano-HPLC unit (Dionex UltiMate 3000 RSLC-nano system; Thermo Fisher Scientific) online coupled to a qTOF mass-spectrometer (Impact II, Bruker-Daltonic) equipped with Data Analysis 4.4 (Bruker Daltonic). A Thermo Scientific Acclaim Pepmap RSLC 100 C18-LC-column (75 µm x 15 cm) in combination with a Thermo Scientific Acclaim Pepmap 100 C18-LC-precolumn (100 µm x 2 cm; both Thermo Fisher Scientific) and gradient elution was used for the chromatographic separation (solvent A. water with 0.1% formic

acid; solvent B, 80% acetonitrile and 0.08% formic acid in water: 0-6 min: 2% B; 6–16 min: 0-65% B; 16–19 min: 65% B; 19–21 min: 65-99% B; 21–24 min: 99% B; flow rate of 0.3 µl/min). The mass spectrometer was operated in positive mode (spray voltage: 4500 V; offset voltage: 500 V; nitrogen dry gas: 4 L min^{-1}; drying temperature: 180°C; nebulizing gas pressure: 0.4 bar). We used an acquisition rate of 2 Hz for MS spectra within the *m*/*z* range 50-2000. In the case of auto MS/MS analyses, the acquisition rate was set at 2 Hz and 10 Hz for low (<25 000) and for high (>25 000) signal intensities, respectively. Line spectra calculated as a sum of intensities were registered and two technical replicates were accumulated per sample. Raw mass-spectrometric data were recalibrated and the extracted ion chromatograms of $[M+3H]^{3+}$ signal for CBF at m/z 748.66 were calculated using data analysis 4.4.

IDENTIFICATION OF POST-TRANSLATIONAL MODIFICATION OF CHROMOGRANIN A CLEAVING ENZYMES BY MATRIX-ASSISTED LASER DESORPTION/IONISATION TIME OF FLIGHT MASS SPECTROMETRY

Post-translational modifications of chromogranin A cleaving enzymes were identified using matrix-assisted laser desorption/ionization-time of flight mass spectrometry (MALDI-TOF/TOF mass spectrometry) as previously described [5]. Briefly, proteins of 35 µg adrenal glands of C57BL/6J $ApoE^{-/-}$ mice (bred and housed under specific pathogen-free conditions in the animal facility of RWTH Aachen university hospital; agreement number: 81-02.04.2017.A504) and C57BL/6J (bred and housed under specific pathogen-free conditions in the animal facility of RWTH Aachen university hospital; agreement number: 81-02.04.2017.A503) with and without chronic kidney disease (CKD) were isolated. In C57BL/6J $ApoE^{-/-}$ mice the induction of CKD was performed by a 1.5 weeks diet of 0.3% adenine. A stable CKD condition was subsequently achieved with 0.15% adenine for 4.5 weeks. In addition, the animal received a high-fat diet during the whole time of experiments starting four weeks before the induction phase. In C57BL/6J mice the induction of CKD was performed by a 1.5 weeks diet of 0.3% adenine followed by a maintenance phase of

6.5 weeks diet of 0.2% adenine. The induction and maintenance of CKD were monitored by analysis of plasma urea and creatinine levels. The glands were then mechanically pulverized and homogenized and an equal amount of proteins were separated by 12% SDS-polyacrylamide gel electrophoresis. Proteins were stained using Coomassie Brilliant Blue G-250 (BioRad, Munich, Germany). Proteins corresponding to sizes of 75-120 kDa were separated and equilibrated using ammonium bicarbonate in acetonitrile. Proteins were trypsinized with ammonium bicarbonate (50 mmol L^{-1}) and 0.03% w/v trypsin for 24 h at 37°C. The resulting tryptic peptides were desalted and concentrated utilizing the $ZipTip_{c18}$ technology (Millipore, Billerica, MA, USA) and water with 0.3% trifluoroacetic acid and eluted with 80% acetonitrile directly onto the MALDI target plate (MTP-Ground steel 400/384; Bruker-Daltonic) using alpha-cyano-4-hydroxycinnamic acid as matrix. The subsequent mass spectrometric (MS) analyses were performed using a MALDI-TOF/TOF mass spectrometer (Ultraflex III; Bruker-Daltonic, Germany). A database search (Swiss-Prot) using the Mascot 2.2 search engine (Matrix Science Inc, Boston, MA) and Bruker Bio-Tool 3.2 software (Bruker Daltonic, Bremen, Germany) was performed with the calibrated and annotated spectra to calculate the peptide mass signal for each entry into the sequence database, compare the experimental MALDI-MS and MALDI-MS/MS dataset, to assign a statistical weight to each peptide match using empirically determined factor [8].

CELL CULTURE AND CALCIFICATION INDUCTION IN HUMAN AORTIC SMOOTH MUSCLE CELLS AND CEMENTOBLASTS

Human aortic smooth muscle cells (hAoSMCs) (Promocell) were cultivated in a smooth muscle cell medium (Promocell). Immortalized murine osteocalcin expressing cementoblasts were cultivated in DMEM low glucose (1g/L) (Gibco) including 5% FCS and 1% penicillin/streptomycin. Both cell types were seeded in 48 well plates (5x 10^3 cells/well) at 80% confluence. Dulbecco's modified Eagle's medium (DMEM) containing 25 mmol L^{-1} glucose supplemented with 2.5% fetal calf serum and 2.8 mmol L^{-1} phosphate were used to induce calcification (calcifying medium, CM). The calcifying medium was supplemented with CBF (max. concentration 100 pmol L^{-1}) or CBF

fragments (100 nmol L^{-1}). As reference medium, DMEM containing 25 mmol L^{-1} glucose supplemented with 2.5% fetal calf serum and 0.9 mmol L^{-1} phosphate were used (non-calcifying medium, NCM). The hAoSMCs were incubated for 7 days and the cementoblasts for 9 days while the incubation medium was replaced after 48 h.

EX VIVO AND CALCIFICATION OF RAT THORACIC AORTIC RINGS

The thoracic aortas of 8-13 weeks old male Wistar rats were gently dissected and were cut into 3-4 mm segments [4]. The endothelial layer of the thoracic aortic ring was manually damaged by scratching using a conventional pipette tip. The thoracic aortic rings were incubated with DMEM containing 25 mmol L^{-1} glucose supplemented with 2.5% fetal calf serum and 2.8 mmol L^{-1} phosphate to induce calcification. The calcification medium was supplemented with CBF (max. concentration 1 µmol L^{-1}) or CBF fragments (100 nmol L^{-1}). As reference medium, DMEM containing 25 mmol L^{-1} glucose supplemented with 2.5% fetal calf serum and 0.9 mmol L^{-1} phosphate were used (non-calcifying medium, NCM). The thoracic aortic rings were incubated for 7 days while the medium was replaced after 48 h.

CO-IMMUNOPRECIPITATION (CO-IP)

5x 10^6 HAoSMCs cells were lysed using 1 ml of lysis buffer including 50 mmol L^{-1} Tris HCl, 1% Triton-X-100, 2% CHAPSO, 100 mmol L^{-1} NaCl and 15 mmol L^{-1} EGTA, supplemented with EDTA-free Halt Protease Inhibitor Cocktail (1:10; Sigma-Aldrich) and Halt Phosphatase Inhibitor Cocktail (1:10; Sigma-Aldrich) for 1 h at 4°C. The cell lysate was incubated with 50 µg CBF-His tag peptide for 1 h at 4°C. The cell lysate was centrifuged at 13,000g at 4°C for 15 min. 100 µl of the supernatant was kept as input. The rest was incubated with either rabbit IgG isotype control antibody (5 µg ml^{-1}, Genetex) or polyclonal rabbit anti-TGFβ-receptor antibody (5 µg ml^{-1}, MyBioSource) or polyclonal rabbit anti-TNFα-receptor antibody (12.5 µg ml^{-1}, RayBiotech), respectively, overnight at 4°C. Immunoprecipitation was performed by Protein A/G magnetic beads for 2 h at 4°C (25 µl per sample). Eluents were resuspended in 100 µL of 1x sample buffer with 100 mmol L^{-1} DTT. All

samples were stored at −80°C for further Western blot analysis. Samples were resolved by 10% SDS–polyacrylamide gel electrophoresis, transferred to nitrocellulose membranes, blocked with 5% bovine serum albumin (BSA) for 1 h at room temperature. The blots were incubated with the polyclonal rabbit anti-TGFβ-receptor antibody (1:1000, Abcam) or monoclonal mouse anti-TNFα-receptor antibody (1:1000, Biorbyt) or polyclonal rabbit anti-His-tag antibody (1:1000, Genetex) at 4°C overnight. The blots were subsequently incubated with horseradish peroxidase-conjugated anti-rabbit immunoglobulin G (IgG; Santa Cruz Biotechnology) or anti-mouse immunoglobulin G (IgG; Santa Cruz Biotechnology) at 1:10,000 for 1 h at room temperature. Immunoreactive bands were visualized via enhanced chemiluminescence, and densitometry was performed using Quantity One software (Bio-Rad Laboratories).

TRANSFECTION OF HUMAN AORTIC SMOOTH MUSCLE CELLS

For siRNA transfection HAoSMCs were washed once with PBS, detached, collected by centrifugation and resuspended in a growth medium. The transfection was performed by the Neon Transfection System (Thermo Fisher Scientific, Germany). The PIT-1 siRNA with sequence 5´-GCCGTAGTTTACAGTATTTAA-3´ was used. A GFP siRNA from Quiagen served as a control. 20 µl of cell suspension in Buffer R, containing approximately 650,000 cells and 120 nmol L^{-1} siRNA was placed in a sterile electroporation tip from the Neon Transfection System. Cells were subjected to high voltage 1,470V at a setting that had been optimized for HAoSMCs. After electroporation, the cells were immediately plated out using a growth medium in 48 well plates 45,000 cells/well. After 24 h the growth medium was replaced with DMEM containing 25 mmol L^{-1} glucose supplemented with 2.5% fetal calf serum and 2.8 mmol L^{-1} phosphate to induce calcification. The calcification medium was supplemented with CBF (100 nmol L^{-1}). As reference medium, DMEM containing 25 mmol L^{-1} glucose supplemented with 2.5% fetal calf serum and 0.9 mmol L^{-1} phosphate were used (non-calcifying medium, NCM). The cells were incubated for 7 days while the medium was replaced after 48 h.

EFFECT OF CBF IN AN ANIMAL MODEL WITH ENHANCED ARTERIAL CALCIFICATION

An animal model of elastocalcinosis with proven increased arterial calcification [9] was chosen to assay the effects of CBF *in vivo*. Animal experiments were approved by the regional ethics committee "Comité d'éthique pour l'expérimentation animale Languedoc Roussillon N°36", with the agreement number APAFIS 2018072715539434#17270v4, and conformed to the Guide for the Care and Use of Laboratory Animals, NIH (National Academies Press US, 8th edition, 2011). Three groups of 6 weeks old Wistar rats (Charles River Laboratories) were given a regular rat chow (A04, Safe, Villemoisson-sur-Orge) and spring water (Mont Roucous®; calcium 60 µmol L^{-1}) *ad libitum* for one week before experiments. Two groups received CBF (31 µg kg^{-1} per day) or its vehicle (saline) infused through an osmotic pump (Alzet 2004, Charles River) implanted under the skin. CBF treatment was initiated 3 or 4 days before induction of elastocalcinosis (vitamin D_3 plus nicotine, or VDN rats) with a single injection of vitamin D_3 (300,000 IU kg^{-1}, i.m.) and two gavages of nicotine (25 mg kg^{-1}, 5 ml kg^{-1}) on the same day, as previously described [3]. Untreated rats served as control rats (sham operation and saline administration). Four weeks later, rats were anaesthetized (ketamine-xylazine), and a catheter was inserted into the right carotid artery for blood pressure measurement. After 10-15 min of equilibration, systolic, diastolic, mean arterial, and pulse (systolic-diastolic) pressures were determined. The aorta was gently dissected, adventitial fat removed, and cut into segments (3-4 mm) for further analysis.

HISTOLOGICAL CALCIFICATION STAINING AND IMMUNOFLUORESCENCE STAINING

Thoracic aortic rings were placed in 1 ml of 4% formaldehyde for 1 h to prepare for histological and immunohistochemical staining. The rings were dehydrated and embedded in paraffin and 5 µm slices were prepared by a microtome (Leica RM 2250). The slices were placed on a glass slide, deparaffinized in xylene, and dehydrated in decreasingly concentrated isopropanol solutions before staining. Von Kossa staining was performed to visualize calcified areas of the aortic rings. The aortic ring slides were incubated with 5% silver nitrate in the presence of UV light at 405 nm for 1 h, washed three times with double-distilled water (ddH_2O), incubated in the presence

of 5% sodium thiosulfate in water for 1 min, and washed three times with ddH_2O. Next, nuclear fast red (Merck) was added to the slides for 5 min. The slides were then rinsed, dehydrated with ethanol, incubated for 5 min in xylol, and covered with Vitro-Clud (R. Langenbrinck).

For immunohistochemical analyses, sections were blocked with blocking solution (1% bovine serum albumin (BSA)) for 30 min at room temperature. The sections were incubated with monoclonal mouse anti-α-SMA antibody 1:200 (Dako), polyclonal rabbit anti-phospho-SMAD1 antibody 1:100 (Biorbyt), or polyclonal rabbit anti-phospho-SMAD5 antibody 1:200 (Biorbyt) at 4°C overnight. Detection was carried out for α-SMA staining with polyclonal goat anti-mouse-Cy3, 1:300 1 h, at room temperature (Jackson ImmonoResearch) and for p-SMAD1 and p-SMAD5 staining with polyclonal goat anti-rabbit-Biotin (Vector Laboratories, Burlingame), 1:100 for 30 min at room temperature, followed by Streptavidin-FITC (Vector Laboratories, Burlingame), 1:30 for 30 min at room temperature. Samples were visualized with a LEICA DM5500B microscope equipped with a digital imaging system. Staining or protein expression was determined by the area stained and expressed as a percentage of the total aortic area section, using ImageJ software (National Institutes of Health) after RGB split and segmentation of the image.

MEASUREMENT OF CALCIUM CONTENT

For the determination of calcium content, cells or thoracic aortic rings were washed 3 times with PBS without calcium. Aortic rings were dried and weighed. Dried aortic rings and cells were decalcified with 0.1 M HCl for 24 h, and the calcium content in the supernatant was determined by the o-cresolphthalein complexone method (Clinical Chemistry Assay Kit/Calcium) according to the manufacturer’s protocol. Cell content was extracted in 0.1 M NaOH 0.1% sodium dodecyl sulphate (SDS), and protein content was measured by the bicinchoninic acid (BCA) protein assay method (Thermo Fisher Scientific). The calcium content was normalized to total protein for cells or dry weight for aortic rings.

QUANTITATIVE POLYMERASE CHAIN REACTION (qPCR) ANALYSES OF mRNA EXPRESSION IN HUMAN AORTIC SMOOTH MUSCLE CELLS AND VDN RATS

Total RNA was extracted from hAoSMCs and aortic tissue using RNAeasy mini kit (Qiagen). Reverse transcription was performed using 1 µg total RNA, random hexamers, and Verso reverse transcriptase (Thermo Scientific) as per the manufacturer's instructions. For real-time PCR, gene expression levels were quantified using SYBR Green I dye chemistry on a LightCycler 480 system (Roche Applied Sciences). PCR primers (**Table 2**) were designed using the LightCycler Probe Design software 2.0. Expression levels were determined with the LightCycler analysis software (version 3.5) relative to standard curves. Data were represented as the mean level of gene expression relative to the expression of the reference gene (β-Actin for cells and RPLPO for rats).

CLINICAL STUDY OF PLASMA CBF LEVELS IN END-STAGE RENAL DISEASE

In the first clinical study, study participants have given written informed consent were recruited from University Hospital Essen (Germany) after approval by the local ethics committee (ethical vote 08–3817). 17 patients suffered from CKD stage 5 (ESRD) and were on regular dialysis. 13 patients had no CKD (controls) with estimated glomerular filtration rates according to the MDRD-formula ≥60 ml min^{-1} 1.73^{-1} m^{-2}. Creatinine and haemoglobin were measured using standard autoanalyzer techniques. Intact parathyroid hormone was determined by immunoassay (Roche Diagnostics). Plasma samples were immediately separated from blood cells and were frozen until all samples were collected, which were measured side by side within one analysis set-up. In the second study, 10 patients who suffered from ESRD were recruited from University Hospital Aachen (Germany) after approval by the local ethics committee (ethical vote EK 218-17).

MEASUREMENT OF THE BONE DENSITY

The bone density was measured from the tibia of untreated control Wistar rats, VDN rats receiving a vehicle infusion (VDN) and VDN rats receiving an infusion of the active site of CBF (CBF01-10) (31µg kg^{-1} per day for 4 weeks) via an osmotic pump. The tibia was isolated from these rats and

fixed with 4% PFA. A CT scan was performed with a resolution of 40 µm/pixel for each tibia and bone density was calculated based on a threshold of 2,000 units.

STATISTICS

Statistical analysis was performed using GraphPad Prism 8 (GraphPad Software, USA), and the data were represented as mean ± SEM. Analysis of variance (ANOVA) for a single factor (one-way) or multiple factors (two-way) was performed to determine differences between treatment groups and the control group. In both, Bonferroni's multiple comparisons were used as a post-test. Differences at $P<0.05$, *$P<0.05$, **$P\leq0.01$, ***$P\leq0.001$, ****$P\leq0.0001$ were considered to be statistically significant.

References

1. Aunis D, Miras-Portugal MT, Mandel P (1974) Bovine adrenal medullary dopamine-beta-hydroxylase: studies on the structure. Biochim Biophys Acta 365:259-273
2. Beyermann M, Fechner K, Furkert J, Krause E, Bienert M (1996) A single-point slight alteration set as a tool for structure-activity relationship studies of ovine corticotropin releasing factor. J Med Chem 39:3324-3330
3. Gaillard V, Jover B, Casellas D, Cordaillat M, Atkinson J, Lartaud I (2008) Renal function and structure in a rat model of arterial calcification and increased pulse pressure. Am J Physiol Renal Physiol 295:F1222-1229 doi:10.1152/ajprenal.00081.2008
4. Holmar J, Noels H, Bohm M, Bhargava S, Jankowski J, Orth-Alampour S (2020) Development, establishment and validation of in vitro and ex vivo assays of vascular calcification. Biochem Biophys Res Commun 530:462-470 doi:10.1016/j.bbrc.2020.05.085
5. Jankowski V, Schulz A, Kretschmer A, Mischak H, van der Giet M, Janke D, Schuchardt M, Herwig R, Zidek W, Jankowski J (2013) The enzymatic activity of the VEGFR2-receptor for the biosynthesis of dinucleoside polyphosphates. J Mol Med 91:1095-1107
6. Jankowski V, Tölle M, Vanholder R, Schönfelder G, van der Giet M, Henning L, Schlüter H, Paul M, Zidek W, Jankowski J (2005) Identification of uridine adenosine tetraphosphate (Up_4A) as an endothelium-derived vasoconstrictive factor. Nature Medicine 11:223-227
7. Klein J, Eales J, Zurbig P, Vlahou A, Mischak H, Stevens R (2013) Proteasix: a tool for automated and large-scale prediction of proteases involved in naturally occurring peptide generation. Proteomics 13:1077-1082 doi:10.1002/pmic.201200493
8. Kork F, Jankowski J, Goswami A, Weis J, Brook G, Yamoah A, Anink J, Aronica E, Fritz S, Huck C, Schipke C, Peters O, Tepel M, Noels H, Jankowski V (2018) Golgin A4 in CSF and granulovacuolar degenerations of patients with Alzheimer disease. Neurology 91:e1799-e1808 doi:10.1212/WNL.0000000000006457

9. Niederhoffer N, Lartaud-Idjouadiene I, Giummelly P, Duvivier C, Peslin R, Atkinson J (1997) Calcification of medial elastic fibers and aortic elasticity. Hypertension 29:999-1006
10. Pintor J, Torres M, Miras-Portugal MT (1991) Carbachol induced release of diadenosine polyphosphates--Ap4A and Ap5A-- from perfused bovine adrenal medulla and isolated chromaffin cells. Life Sci 48:2317-2324
